# *Strongyloides stercoralis*: Global Distribution and Risk Factors

**DOI:** 10.1371/journal.pntd.0002288

**Published:** 2013-07-11

**Authors:** Fabian Schär, Ulf Trostdorf, Federica Giardina, Virak Khieu, Sinuon Muth, Hanspeter Marti, Penelope Vounatsou, Peter Odermatt

**Affiliations:** 1 Department of Epidemiology and Public Health, Swiss Tropical and Public Health Institute, Basel, Switzerland; 2 University of Basel, Basel, Switzerland; 3 National Center for Parasitology, Entomology and Malaria Control, Ministry of Health, Phnom Penh, Cambodia; 4 Medical and Diagnostics Department, Swiss Tropical and Public Health Institute, Basel, Switzerland; London School of Hygiene & Tropical Medicine, United Kingdom

## Abstract

**Background:**

The soil-transmitted threadworm, *Strongyloides stercoralis*, is one of the most neglected among the so-called neglected tropical diseases (NTDs). We reviewed studies of the last 20 years on *S. stercoralis's* global prevalence in general populations and risk groups.

**Methods/Principal Findings:**

A literature search was performed in PubMed for articles published between January 1989 and October 2011. Articles presenting information on infection prevalence were included. A Bayesian meta-analysis was carried out to obtain country-specific prevalence estimates and to compare disease odds ratios in different risk groups taking into account the sensitivities of the diagnostic methods applied. A total of 354 studies from 78 countries were included for the prevalence calculations, 194 (62.4%) were community-based studies, 121 (34.2%) were hospital-based studies and 39 (11.0%) were studies on refugees and immigrants. World maps with country data are provided. In numerous African, Asian and South-American resource-poor countries, information on *S. stercoralis* is lacking. The meta-analysis showed an association between HIV-infection/alcoholism and *S. stercoralis* infection (OR: 2.17 BCI: 1.18–4.01; OR: 6.69; BCI: 1.47–33.8), respectively.

**Conclusions:**

Our findings show high infection prevalence rates in the general population in selected countries and geographical regions. *S. stercoralis* infection is prominent in several risk groups. Adequate information on the prevalence is still lacking from many countries. However, current information underscore that *S. stercoralis* must not be neglected. Further assessments in socio-economic and ecological settings are needed and integration into global helminth control is warranted.

## Author Summary

The soil-transmitted threadworm *Strongyloides stercoralis* is one of the most neglected helminth infections. It is endemic world-wide, yet more prevalent in hot and humid climates as well as resource poor countries with inadequate sanitary conditions. The difficult diagnosis and irregular excretion of larvae lead to an underreporting of infection rates. We reviewed the literature of the last 20 years reporting on infection rates of *S. stercoralis*. Including the sensitivity of diagnostic methods applied, we modeled and mapped for the first time country-wide prevalence estimates. The modeling was divided into studies reporting infection rates in the general population, in hospitals and on refugees & immigrants, respectively. We further summarized possible risk factors for *S. stercoralis* infection using meta-analysis. The most prominent risk factors include HIV-infection, HTLV-1 infection and alcoholism. Information on infection rates is missing in many countries. Our results show high prevalence estimates in many resource poor tropical and subtropical countries. We conclude that *S. stercoralis* should not be neglected and that further studies applying high sensitivity diagnostic methods are needed.

## Introduction

The threadworm *Strongyloides stercoralis* is a soil-transmitted nematode and one of the most overlooked helminth among the neglected tropical diseases (NTDs) [1]. It occurs almost world-wide, excluding only the far north and south, yet estimates about its prevalence are often little more than educated guesses [2], [3]. Compared to other major soil-transmitted helminths (STHs), namely *Ascaris lumbricoides* (roundworm), *Necator americanus* and *Ancylostoma duodenale* (hookworms) and *Trichuris trichiura* (whipworm), information on *S. stercoralis* is scarce [3]. The diagnostic methods most commonly used for STH detection, such as direct fecal smear or Kato-Katz, have low sensitivity for *S. stercoralis* or fail to detect it altogether [4]–[6]. Especially the parasitological diagnostic tools for *S. stercoralis* infection like the Koga Agar plate culture consume more resources and time than the most commonly applied methods [7] and hence, are rarely used in potentially endemic settings of resource poor countries.

**Figure 1:**
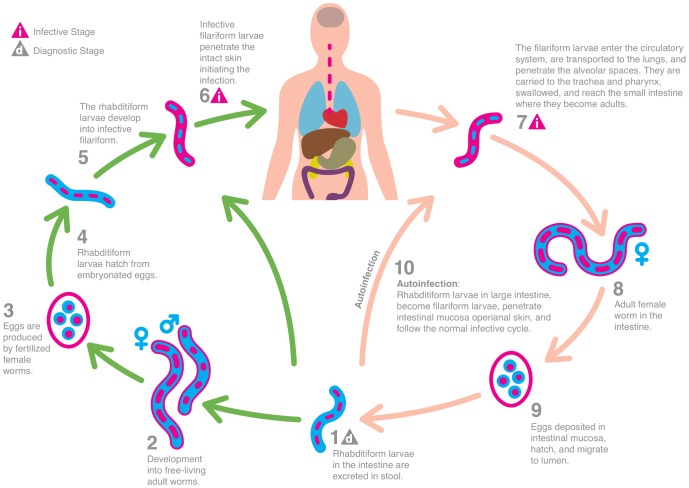
The life-cycle of *S. stercoralis* (based on http://www.dpd.cdc.gov/dpdx).

*S. stercoralis* was first described in 1876. The full life cycle, pathology and clinical features in humans were fully disclosed in the 1930s (Figure 1). The rhabditiform larvae are excreted in the stool of infected individuals. The larvae mold twice and then develop into infective 3^rd^ stage filariform larvae (L_3_), which can infect a new host by penetrating intact skin. The larvae thrive in warm, moist/wet soil. Walking barefoot and engaging in work involving skin contact with soil, as well as low sanitary standards are risk factors for infection. Hence, many resource poor tropical and subtropical settings provide ideal conditions for transmission [8]–[10].

*S. stercoralis* is an exception among helminthic parasites in that it can reproduce within a human host (endogenous autoinfection), which may result in long-lasting infection. Some studies report individuals with infections sustained for more than 75 years [9]–[13]. Two other species, closely related to *S. stercoralis*, also infect humans, namely *S. fulleborni* and *S. cf fulleborni*, which are of minor importance and geographically restricted [14], [15].

*S. stercoralis'* ability to cause systemic infection is another exceptional feature of the threadworm. Particularly in immunosuppressed individuals with a defective cell-mediated immunity, spread from the intestinal tract of one or more larval stages may lead to hyperinfection syndrome and disseminated strongyloidiasis, in which several organs may be involved [16]. The outcome is often fatal [5], [17], [18]. In contrast, uncomplicated intestinal strongyloidiasis may include a spectrum of unspecific gastro-intestinal symptoms such as diarrhea, abdominal pain and urticaria [16], [19].

However, most infections, chronic low-intensity infections in particular, remain asymptomatic. Asymptomatic infections are particularly dangerous. In cases of immunosuppressive treatment, especially with corticosteroids, they have the potential to develop fatal disseminated forms. Proper screening of potentially infected individuals before immunosuppressive treatment (coprologically over several days and/or serologically) is essential, though often not carried out. This asymptomatic infection, coupled with diagnostic difficulties, (often due to irregular excretion of parasite larvae) leads to under-diagnosis of the threadworm. Assessing the clinical consequences of infection remains challenging, thus, little is known about the *S. stercoralis* burden in endemic countries.

In 1989, Genta [2] summarized information on global distribution of this parasite for the first time. He found *S. stercoralis* to be highly prevalent in Latin America and sub-Saharan Africa. He further pointed out that many reports suggested high infection rates in South-East Asia and described several risk groups, including refugees and immigrants.

The objectives of our study are to obtain country-wide estimates of *S. stercoralis* infection risk in the general population, and to assess the association between *S. stercoralis* prevalence and different risk groups. We reviewed the available literature and carried out a Bayesian meta-analysis taking into account the sensitivity of the different diagnostic tools. The models allowed estimation of the diagnostic sensitivity for different study types and risk groups.

## Materials and Methods

### Literature search and data extraction

We conducted a systematic literature review of all research papers published between January 1989 and October 2011 and listed in PubMed. Papers were filtered using the search terms “Strongyloides” or “Strongyloides stercoralis” or “Strongyloidiasis”. Studies were included if they contained information on prevalence and/or risk of *S. stercoralis* infection, either in the general population or in risk groups, i.e. patients with HIV/AIDS, immuno-deficiencies, HTLV-1-infection, alcoholism, and diarrhea.

We excluded articles (i) that were not written in English, Spanish, Portuguese, French or German language; (ii) that referred to specific bio-molecular research aspects of *S. stercoralis*; (iii) on infection in animals, and (iv) that did not provide additional information on the prevalence and/or risk of *S. stercoralis* infection.

For each selected paper, the following information was recorded: number of infected individuals, number of examined individuals, risk factors (specific risk group or control group), study area (country or geographic coordinates, when available) and WHO world region (Region of the Americas, European region, African region, Eastern Mediterranean region, South East Asia region and the Western Pacific region), study type (cross-sectional, case-control etc.), place of implementation (community- or hospital-based studies, and studies on refugees and immigrants), and diagnostic procedures used (copro-diagnostic, serological methods etc.).

### Statistical analysis

The main outcome of the analysis is *S. stercoralis* prevalence in the general population for each country as well as in specific risk groups, namely HIV/AIDS patients, HTLV-1 patients, alcoholics and patients with diarrhea.

A Bayesian model for meta-analysis that included the diagnostic-test sensitivity was formulated and implemented in WinBUGS 1.4 [20].

Information about the sensitivity of the different diagnostic tools used was derived from the literature and led to the division of diagnostic procedures into three sensitivity groups. We assigned a range of sensitivity using the lowest and the highest sensitivity reported, respectively [21]–[43]. The three groups are as follows: (i) copro-diagnostic procedures with low sensitivity (12.9–68.9%); (ii) copro-diagnostic procedures with moderate sensitivity (47.1–96.8%); (iii) serological diagnostic procedures with high sensitivity (68.0–98.2%). Beta prior distributions were specified for the different diagnostic-test group sensitivities. A more detailed description of the prior elicitation can be found in the appendix.

#### Estimating country-wide prevalence in the general population

The retrieved data was analyzed separately in the three different subsets: community-based studies, hospital-based studies, and studies on refugees and immigrants, as prevalence rates from these subsets cannot be directly compared.

Model-based prevalence estimates for each study type and country were plotted on a world map, using ArcGIS (version 9.3). The prevalence estimates for refugee and immigrant studies were displayed in the country where the study was undertaken and not in the country from where the refugees and immigrants originated. Further details regarding model specification can be found in appendix.

#### Association with specific risk factors

To analyze the association between *S. stercoralis* and specific risk factors, namely HIV/AIDS, Human T-lymphotropic virus 1 (HTLV-1) infected individuals, diarrhea, and alcoholism, the studies were grouped into case-control studies and cross-sectional studies. We used case-control studies conducted on each risk group with complete information about individuals screened (tested) and infected with *S. stercoralis*, as well as the diagnostic method used, to model specific Odds Ratios (OR) and pool them into an overall estimate using a logistic model taking into account the prior information available on diagnostic test sensitivity. In the appendix, we describe the formulation of the Bayesian model for OR estimations of the risk factors mentioned above. The same model without the inclusion of the sensitivity was implemented and run. Results are shown, for comparison purposes, in the appendix (Figure A1a–A1d). Forest plots were produced using R software (version 2.13.1).

#### Diagnostic test sensitivity

The Bayesian models employed in this study estimate the disease prevalence (or ORs) together with the diagnostic sensitivity. We run the models under different prior specifications, to assess the robustness of the estimates.

## Results

### Study identification

**Figure 2:**
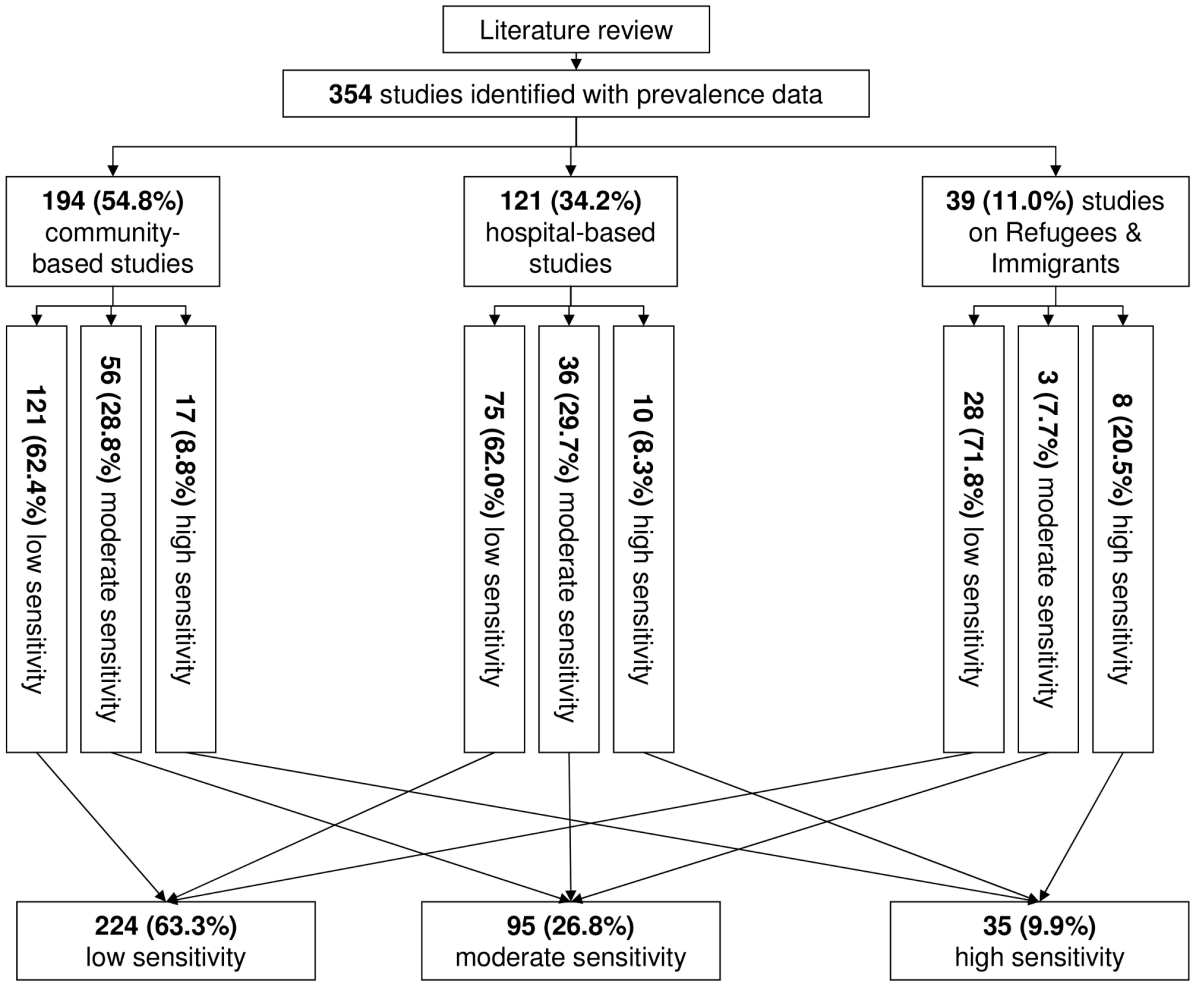
Flowchart of the literature review.

We identified and reviewed 354 studies (Figure 2). Of those, 194 (54.8%) used a cross-sectional design and were conducted in communities: 121 (62.4%) used diagnostic methods with low sensitivity, 56 (28.9%) with moderate sensitivity, and 17 (8.8%) with high sensitivity. Out of 121 hospital-based studies, 75 (61.5%) used low, 36 (29.8%) used moderate and 10 (8.3%) used high sensitivity methods. Of the 39 studies on refugees and immigrants, 28 (71.8%) used low, three (7.7%) used moderate, and eight (20.5%) used high sensitivity diagnostic methods.

### Prevalence

#### Available information

**Figure 3:**
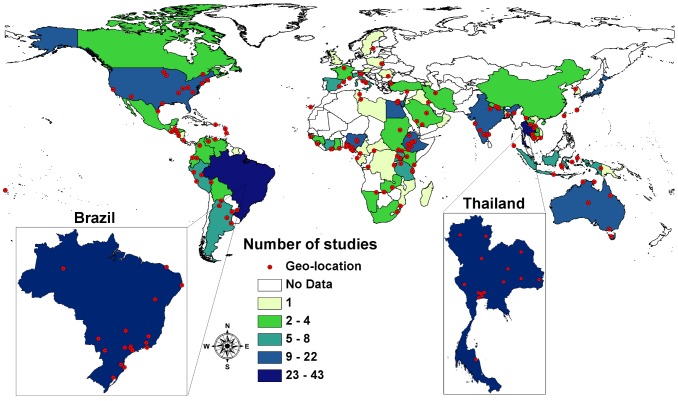
Number of studies undertaken per country since 1989, with geo-location if indicated; Thailand and Brazil displayed separately.

Figure 3 indicates the number of reports per country that provided information on infection rates. Tables 1–3 report the calculated prevalence rates per country. Information is notably scarce for those African countries where environmental and socioeconomic conditions are most favorable for transmission. *S. stercoralis* infection data is only available for 20 (43.5%) of the 46 African countries. The distribution of infection rate information is heterogeneous. Almost a quarter of the studies (18, 23.4%) were undertaken in densely populated Nigeria alone. Some studies reported on tropical West and East Africa. However, infection rate data is scarce for Sahelian, Central and Southern Africa. Most of the available studies used low sensitivity diagnostic methods. Adequate diagnostic techniques, such as the Baermann method and Koga Agar plate culture, were employed in only 19.0% of the studies in African countries.

**Table 1:** Country-wide prevalence rates for *Strongyloides stercoralis* in countries A–F, divided by type of study.

		References	Community-based surveys			Hospital-based surveys			Refugees & Immigrants		
Country	Total Number of surveys for prevalence calculation		Total Number	Prevalence	95% CI	Total Number	Prevalence	95% CI	Total Number	Prevalence	95% CI
Argentina	8	w^1–8^	4	52.8%	42.42%–64.6%	4	63.0%	53.6%–72.9%			
Australia	15	w^9–22^	6	15.0%	13.52%–16.8%	3	28.9%	26.2%–31.6%	6	25.3%	22.3%–28.5%
Austria	1	w^23^				1	5.2%	1.0%–15.7%			
Bangladesh	1	w^24^	1	29.8%	21.7%–39.8%						
Belize	1	w^25^	1	7.7%	3.3%–14.8%						
Bolivia	2	w^26, 27^	2	21.1%	11.2%–36.1%						
Brazil	43	w^28–70^	26	13.0%	12.0%–14.2%	16	17.0%	15.8%–18.2%	1	35.0%	9.8%–85.4%
Burundi	2	w^71^	1	1.9%	0.4%–5.6%	1	21.6%	11.2%–36.4%			
Cambodia	4	w^72–75^	3	17.5%	15.7%–19.6%	1	13.9%	12.1%–16.0%			
Cameroon	1	w^76^	1	10.0%	3.6%–21.2%						
Canada	3	w^77–79^							3	67.5%	61.3%–73.5%
Central African Republic	2	w^80^	1	0.1%	0.0%–1.2%	1	1.9%	0.4%–5.5%			
China	4	w^81–84^	1	14.0%	9.0%–20.4%				3	17.1%	15.2%–19.2%
Colombia	2	w^85, 86^	1	56.2%	48.0%–65.7%	1	20.2%	6.7%–45.1%			
Costa Rica	1	w^87^	1	6.9%	2.9%–13.6%						
Côte d'Ivoire	5	w^88–92^	4	24.3%	20.7%–28.4%	1	67.7%	41.4%–98.7%			
DR of the Congo	1	w^93^				1	32.7%	20.6%–48.6%			
Dominica	1	w^94^	1	97.6%	78.4%–100%						
Ecuador	2	w^95, 96^	2	27.1%	19.3%–36.9%						
Egypt	12	w^97–108^	5	2.5%	2.0%–3.2%	7	11.1%	9.4%–13.1%			
Ethiopia	12	w^109–120^	7	15.9%	14.1%–17.9%	5	31.0%	23.6%–40.0%			
Fiji	1	w^121^	1	9.3%	2.5%–23.1%						
France	2	w^122, 123^				1	31.1%	22.7%–40.7%	1	5.6%	3.7%–8.9%

**Table 2:** Country-wide prevalence rates for *Strongyloides stercoralis* for countries G-M, divided by type of study.

		References	Community-based surveys			Hospital-based surveys			Refugees & Immigrants		
Country	Total Number of surveys for prevalence calculation		Total Number	Prevalence	95% CI	Total Number	Prevalence	95% CI	Total Number	Prevalence	95% CI
Gabon	1	w^124^	1	91.8%	44.6%–100.0%						
Ghana	2	w^125, 126^	1	69.5%	63.2%–76.9%	1	13.6%	1.1%–53.4%			
Grenada	1	w^127^	1	3.3%	0.3%–13.0%						
Guadeloupe	2	w^128, 129^	1	18.7%	14.5%–23.5%	1	8.3%	7.7%–8.9%			
Guatemala	1	w^130^				1	2.0%	1.5%–2.6%			
Guinea	2	w^131, 132^	2	43.8%	34.7%–54.9%						
Guinea-Bissau	2	w^133, 134^	1	23.7%	18.3%–30.0%	1	84.2%	42.1%–100.0%			
Haiti	1	w^135^	1	1.0%	0.5%–1.8%						
Honduras	4	w^136–139^	1	3.2%	1.5%–6.2%	3	29.8%	24.1%–36.0%			
India	14	w^140–153^	5	6.6%	4.4%–9.4%	9	11.2%	8.6%–14.4%			
Indonesia	6	w^154–159^	6	7.6%	6.2%–9.3%						
Iran	3	w^160–162^	1	0.3%	0.1%–0.5%	2	0.6%	0.1%–1.7%			
Iraq	1	w^163^				1	24.2%	14.1%–38.1%			
Israel	3	w^164–166^	1	94.9%	86.4%–100.0%				2	31.0%	27.0%–35.1%
Italy	5	w^167–171^				4	1.8%	1.4%–2.3%	1	3.3%	0.6%–9.6%
Jamaica	3	w^172–174^	2	27.1%	22.8%–32.1%	1	1.8%	0.9%–3.2%			
Japan	14	w^63, 175–186^	9	18.7%	17.4%–20.4%	5	13.6%	12.7%–14.5%			
Jordan	1	w^187^	1	0.03%	0.0%–0.1%						
Kenya	4	w^188–191^	2	80.2%	61.1%–99.4%	2	7.8%	5.0%–11.5%			
Kuwait	1	w^192^				1	16.3%	14.1%–18.7%			
Lao PDR	4	w^193–196^	3	26.2%	22.5%–30.4%	1	55.8%	37.0%–81.4%			
Libya	1	w^197^							1	1.1%	0.1%–4.5%
Madagascar	1	w^198^				1	52.2%	42.6%–61.6%			
Martinique	2	w^199, 200^	1	3.8%	3.3%–4.4%	1	9.6%	8.1%–11.4%			
Mexico	2	w^201, 202^	1	1.6%	0.2%–6.3%	1	5.7%	1.1%–16.5%			
Mozambique	1	w^203^				1	6.2%	2.5%–12.1%			

**Table 3:** Country-wide prevalence rates for *Strongyloides stercoralis* for countries N-Z, divided by type of study.

		References	Community-based surveys			Hospital-based surveys			Refugees & Immigrants		
Country	Total Number of surveys for prevalence calculation		Total Number	Prevalence	95% CI	Total Number	Prevalence	95% CI	Total Number	Prevalence	95% CI
Namibia	3	w^204–206^	2	99.3%	92.2%–100.0%	1	14.3%	11.6%–17.6%			
Nepal	3	w^207–209^	1	22.8%	10.1%–43.4%	2	5.8%	2.5%–11.2%			
Nicaragua	1	w^210^	1	2.0%	0.6%–4.5%						
Nigeria	18	w^211–229^	13	48.1%	43.3%–53.8%	5	17.6%	15.2%–20.3%			
Occ. Palestinian Territ.	1	w^230^				1	4.2%	0.4%–16.7%			
Oman	1	w^231^	1	3.0%	0.6%–8.7%						
Papua New Guinea	1	w^232^	1	99.0%	90.0%–100.0%						
Peru	6	w^233–238^	4	75.3%	70.8%–82.0%	2	69.3%	61.1%–77.9%			
Puerto Rico	2	w^239, 240^	1	16.0%	3.0%–47.5%	1	33.5%	13.7%–66.6%			
Republic of Korea	2	w^241, 242^	2	0.1%	0.0%–0.1%						
Romania	1	w^243^				1	48.8%	31.1%–72.1%			
Saint Lucia	1	w^244^	1	58.5%	44.1%–76.4%						
Saudi Arabia	3	w^245–247^				1	12.5%	3.3%–31.2%	2	7.1%	5.5%–9.0%
Sierra Leone	3	w^248–250^	3	27.4%	21.5%–34.4%						
South Africa	2	w^251, 252^	2	27.5%	21.3%–34.7%						
Spain	5	w^253–257^	1	14.8%	10.3%–20.3%	1	1.9%	1.6%–2.2%	3	4.2%	2.8%–6.1%
Sudan	3	w^258–260^	2	3.7%	1.9%–6.4%				1	98.9%	89.2%–100.0%
Suriname	1	w^261^	1	63.2%	50.3%–78.2%						
Sweden	1	w^262^							1	1.0%	0.4%–2.1%
Thailand	40	w^63,263–300^	32	23.7%	21.8%–26.1%	8	34.7%	31.6%–38.3%			
Tunisia	1	w^301^				1	0.5%	0.3%–0.9%			
Turkey	3	w^302–304^	1	0.6%	0.4%–0.8%	2	4.1%	2.1%–7.2%			
Uganda	6	w^305–310^	4	19.3%	17.1%–21.9%	2	30.3%	25.1%–36.5%			
UK	1	w^311^	1	12.7%	11.1%–14.5%						
UR of Tanzania	8	w^312–317^	4	7.9%	6.6%–9.5%	4	9.3%	6.1%–13.7%			
US of America	22	w^318–337^	3	2.7%	2.4%–3.0%	5	49.2%	0.1%–99.9%	14	40.4%	37.8%–43.0%
Venezuela	3	w^338–340^	1	2.3%	0.2%–9.1%	2	48.4%	0.2%–99.8%			
Viet Nam	1	w^341^	1	0.02%	0.0%–0.3%						
Zambia	3	w^342–344^	1	6.6%	1.3%–19.4%	2	50.6%	0.2%–99.9%			

The Americas are well covered, with studies in 21 (60.0%) of the 35 countries in this region. Data is mostly missing for smaller countries, such as the Caribbean island nations (Antigua, Barbuda, Bahamas, Barbados, etc.). A large amount of information is available for Brazil, where 43 (37.4%) studies were undertaken. Most investigations were conducted in communities (26, 60.5%) rather than in hospitals (16, 37.2%). For the United States of America, 22 (19.1%) studies were identified. Almost two thirds of them (14, 63.6%) focused on refugees and immigrants.

For Europe, comparably fewer reports (24) were found. Most of them focused on refugees, immigrants and travelers. South-East Asia and the Western Pacific region are reasonably represented, with 40 investigations conducted in Thailand (36.4%), 15 in Australia (13.6%), 14 in Japan (12.7%), and 14 in India (12.7%). Yet, in many other Asian countries where high prevalence of *S. stercoralis* is likely to occur, information on infection rates is limited, and studies often lack the use of high sensitivity methods.

#### Global prevalence of *S. stercoralis*

**Figure 4:**
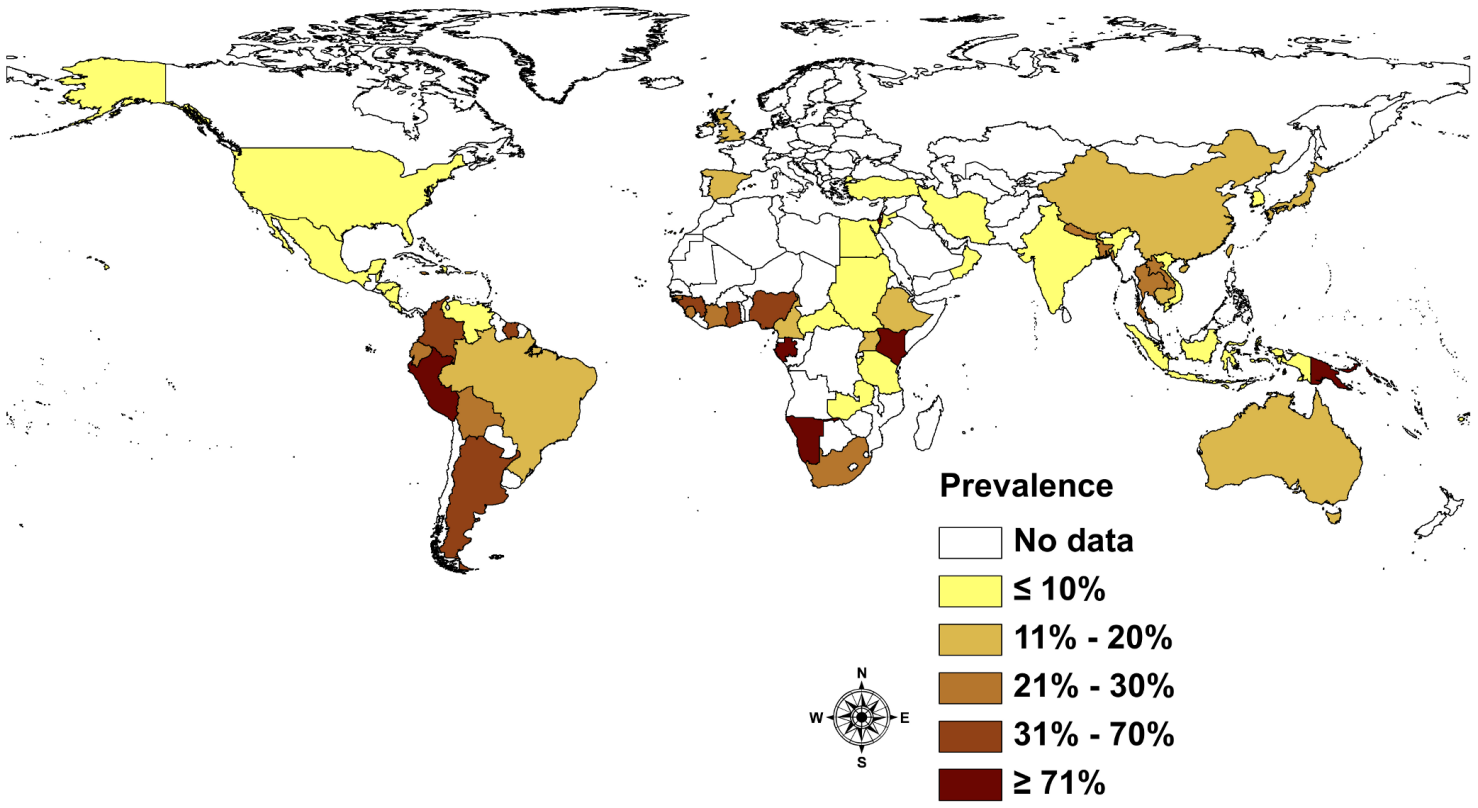
Prevalence of *S. stercoralis* infection by country based community-based studies.

The global prevalence picture is as diverse and heterogeneous as the type and number of studies undertaken. The existing information suggests that *S. stercoralis* infections affect between 10% and 40% of the population in many tropical and subtropical countries. In resource-poor countries with ecological and socioeconomic settings conducive to the spread of *S. stercoralis*, high infection rates of up to 60% can be expected. The majority of the studies reviewed were undertaken at community-level (Figure 4). Yelifari and colleagues [44] conducted one of the biggest studies in Africa, in Northern Ghana, sampling 20,250 persons across 216 villages and therefore covering different settings. The infection rate was 11.6%. They found a slightly higher statistically significant infection rate in men (12.7%) than women (10.6%).

**Figure 5:**
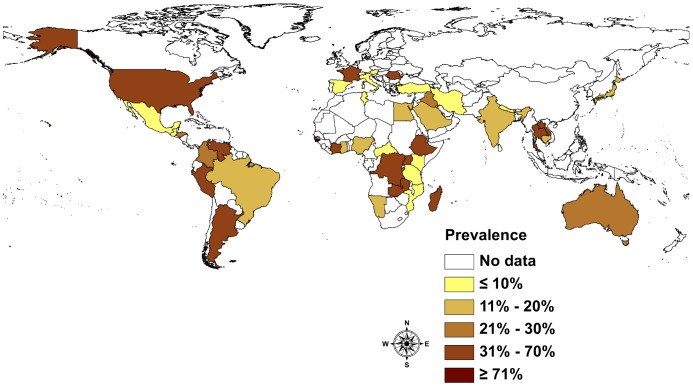
Prevalence of *S. stercoralis* infection by country based on health services studies.

Studies based on health services data often focus on the number of patients reporting symptoms or suffering from conditions other than helminthiasis. If stool samples are analyzed, high sensitivity methods are only applied if the patient is suspected of having an intestinal parasitic infection, i.e. might be infected with *S. stercoralis*. A study from Guadeloupe [45] analyzed 17,660 hospital records from the university hospital in Pointe-à-Pitre, reporting 708 cases of *S. stercoralis* (4.0%). Yet in Guatemala, where 14,914 pregnant women were tested using a single stool sample and where low-sensitivity diagnostic methods were applied, the reported prevalence was as low as 0.4% [46]. This is an example for the difficulties comparing studies using different diagnostic approaches (Figure 5).

**Figure 6:**
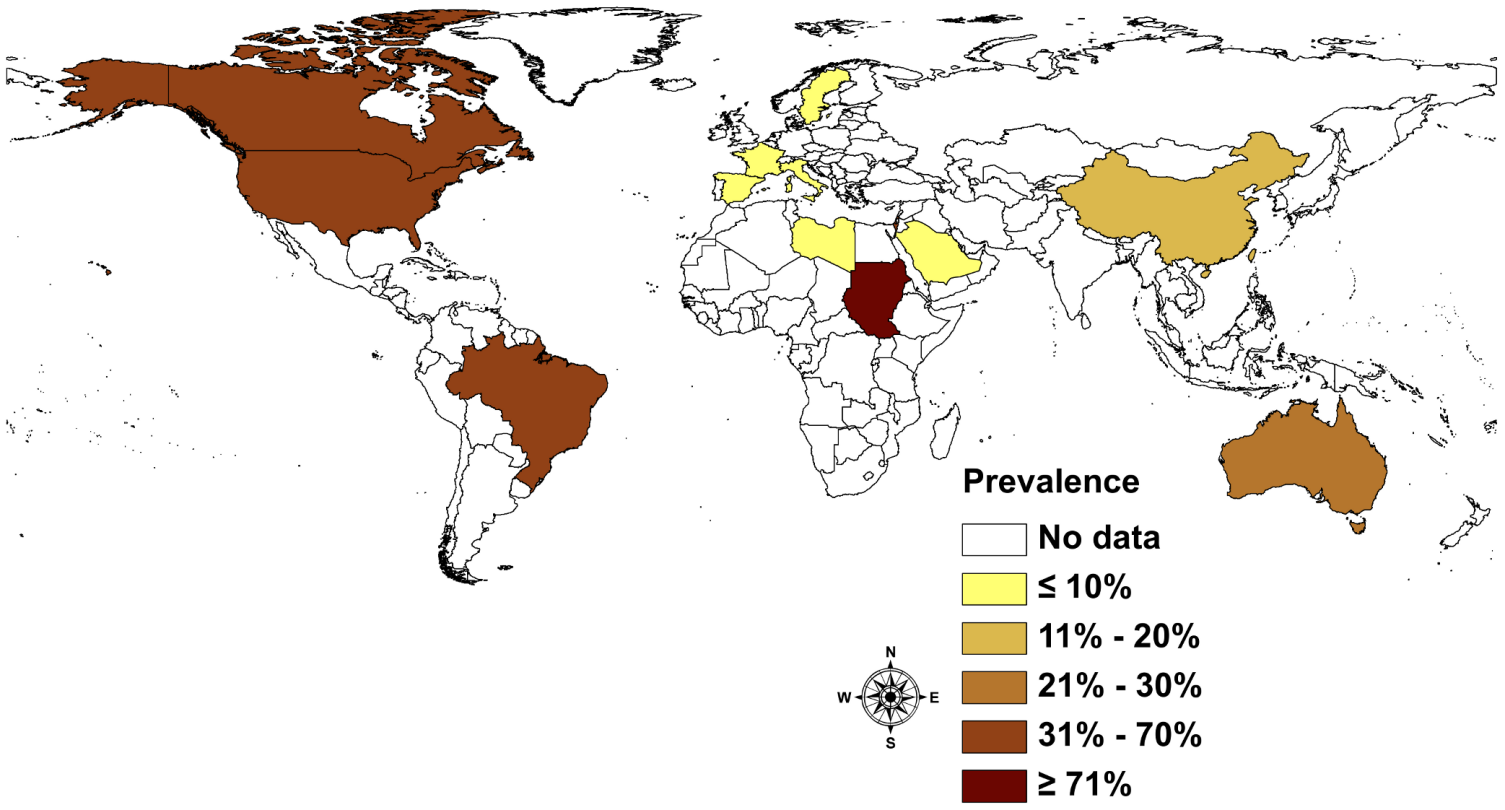
Prevalence of *S. stercoralis* in refugees and immigrants by country.

Studies on refugees and immigrants were mostly conducted, with a few exceptions, in developed countries (Figure 6). Most found high infection rates in immigrants and refugees, reaching prevalence rates of up to 75%. Infection rates varied substantially depending on the refugees' country of origin. In Canada in 1990, Gyorkos and colleagues [40] used high sensitivity diagnostic tools and found a prevalence rate of 11.8% in Vietnamese refugees versus 76.6% in Cambodian refugees. In many countries, immigrants are routinely screened for helminthiasis if they attend a hospital. A study in Saudi Arabia by al-Madani and colleagues [47] analyzed 5,518 female housekeepers originating from different Asian countries. The overall prevalence reported was 0.6%; 0.4% in Filipinos, 0.5% in Indonesians, 1.5% in Sri Lankans, 2.6% in Indians and 3.4% in Thais, respectively.

#### Hotspots: Brazil and Thailand

Brazil and Thailand are *S. stercoralis* endemic countries where reliable and consistent data on infection is available. For Brazil, we found 43 studies (12.1% of all studies world-wide) that qualified for inclusion. Using data from the community-based studies, our model showed a prevalence of 13.0% (95% Bayesian Confidence Interval (BCI): 12.0–14.2%). The Baermann method was used in nine (34.6%) of these studies, and the Koga Agar plate culture in just four (15.4%). Analyzing data from the 16 hospital-based studies yielded a prevalence of 17.0% (95% BCI: 15.8–18.2%). The Baermann method was used in 15 (93.8%) studies, most often in combination with other methods, yet the Koga Agar plate culture was not used in any of the hospital-based studies in Brazil. Most hospital-based studies were undertaken in the big cities of Rio de Janeiro and São Paolo. Rossi and colleagues [48] reported analyzing 37,621 laboratory specimens over a period of two years in the university hospital in the Campinas City region. The patients examined originated from all over Campinas City. The infection rate was estimated to be 10.8%.

In Thailand, a quarter to a third of the study participants tested positive for *S stercoralis.* In all studies conducted directly in the community, the overall prevalence was 23.7% (95% BCI: 21.8–26.1%). In contrast to Brazil, the main diagnostic approach used for the Thai studies was the Koga Agar plate culture, which was used in 10 (31.3%) of the studies. In hospitals (8, 20.0%), the infection prevalence was considerably higher and reached 34.7% (95% BCI: 31.6–38.3%). Five (62.5%) of these studies were undertaken in the capital Bangkok, four of which (50.0%) focused on HIV/AIDS-infected patients.

#### Other regional highlights and concerns

For Japan, all 14 studies were undertaken on the Okinawa islands. *S. stercoralis* is only endemic in Okinawa prefecture and the cases reported are mostly among older persons with sustained infection due to auto-infection. This was demonstrated in a study of Arakaki and colleagues [49] which showed an overall infection rate of 16.4%; yet for individuals aged 10–39 years, the prevalence was only 5.5% whereas in individuals older than 40 years of age, the prevalence was 30.2%. Our country estimate of infection rates based on community data was 18.7% (95% BCI: 17.4–20.4%) and 13.6% (95% BCI: 12.7–14.5%) based on hospital investigations. All the studies from Japan employed a highly sensitive Koga Agar plate culture diagnostic method and often analyzed several stool samples per person. Arakaki and colleagues [50] undertook a study of six different endemic regions in Okinawa, and reported a significant difference between infection rates in males (14.0%) and females (6.8%).

European studies principally focused on refugees, immigrants and travelers to endemic countries. A good example of this is found in a recent report on two Italian tourists returning from Southeast-Asia, presenting acute strongyloidiasis [51]. As an exception, in a study from Spain [52], infections were reported in farm workers in Gandia (south of Valencia, eastern Spain). The Koga Agar plate culture was used on three stool samples taken on consecutive days to diagnose a threadworm infection. Of the 250 farm workers, 12.4% were *S. stercoralis* positive. When adjusted for the sensitivity of the diagnostic method, our model found a prevalence of 14.8% (95% BCI: 10.3–20.3%). Another study from Gill and colleagues [53] of World War II veterans undertaken in 2004 in the United Kingdom showed that *S. stercoralis* infection might be sustained over a long time. Most participants had not left the UK since returning from their deployment in Southeast Asia and were evaluated some 60 years later. The study reported 248 cases from 2,072 veterans screened for *S. stercoralis* (12.0%); the adjusted prevalence was 12.7% (95% BCI: 11.1–14.5%).

Little information is available from countries with the largest populations, namely China and India. Studies on Mainland China are scarce or could not be included due to the language limitations of this review. Our calculation from a study of communities in Yunnan province resulted in a prevalence of 14.0% (95% BCI: 9.0–20.4%). The three other studies identified were conducted on immigrants, mainly from South-East Asian countries, working in Taiwan and presented an infection prevalence of 17.1% (95% BCI: 15.2–19.2%). For India, 14 studies were identified, nine of which were conducted on hospitalized persons, and reporting an infection rate of 11.2% (95% BCI: 8.6–14.4%). Five of these reports focus on HIV/Aids patients. For the five community-level studies, an infection rate of 6.6% was reported (95% BCI: 4.4–9.4%). For other countries with large populations, such as Indonesia, Pakistan and Bangladesh, which combined account for over half a billion inhabitants, only seven studies were available (Indonesia: 6, Bangladesh: 1, Pakistan: 0). All seven studies were conducted at community-level, and infection rates of 7.6% (95% BCI: 6.2–9.3%) in Indonesia and 29·8% (95% BCI: 21.7–39.8%) in Bangladesh, respectively, suggest a considerable burden of infection in these populous countries.

### High risk groups for *Strongyloides stercoralis* infection

#### HIV/AIDS patients

**Figure 7:**
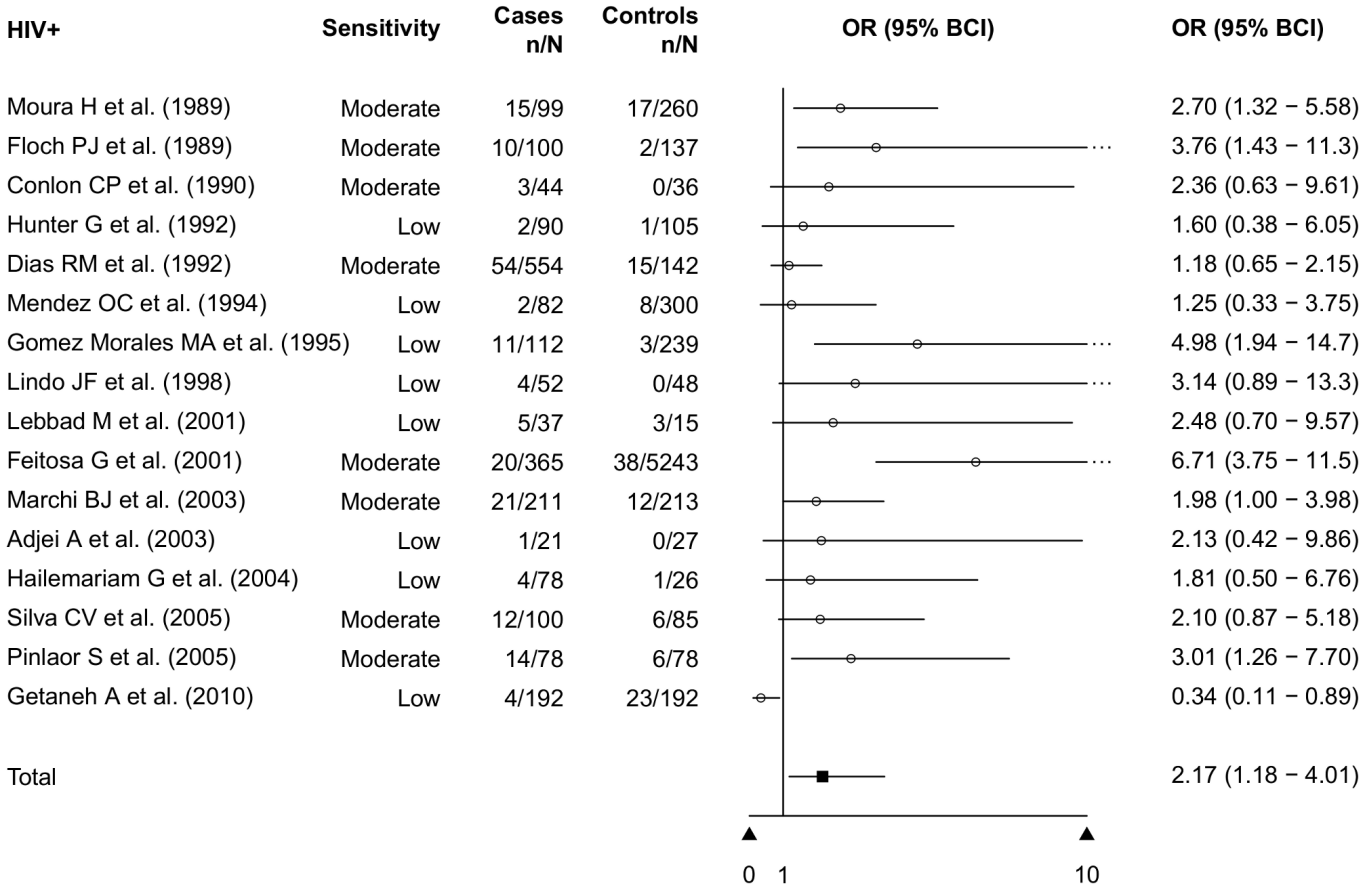
Risk of *S. stercoralis* infection in HIV/AIDS patients (meta-analysis of case-control studies).

Many countries with high HIV-prevalence rates are also highly *S. stercoralis* endemic, and co-infection may occur. *S. stercoralis* no longer constitutes an AIDS-defining, opportunistic infection [54] as it did during the onset of the HIV-pandemic. For 29 cross-sectional studies focusing on HIV-positive individuals, we calculated *S. stercoralis* prevalence rates per country. The rates varied substantially from 1.0% (95% BCI: 0.0–2.0%) in Iran to as high as 43.0% (95% BCI: 20.0–83.0%) in Ethiopia. The overall prevalence for HIV-positive individuals was 10.0% (95% BCI: 5.0–20.0%). We identified 16 case-control studies comparing HIV-positive individuals with sero-negative controls. Four reported a lower or similar prevalence in the two groups [55]–[58]. All other studies showed an increased *S. stercoralis* infection risk for HIV-positive individuals; three showed a statistically significant risk [59]–[61]. Our meta-analysis resulted in a pooled OR of 2.17 (95% BCI: 1.18–4.01) for HIV-positive individuals [28], [55], [56], [58]–[70] (Figure 7) compared to the HIV-negative controls.

#### HTLV-1 patients

**Figure 8:**
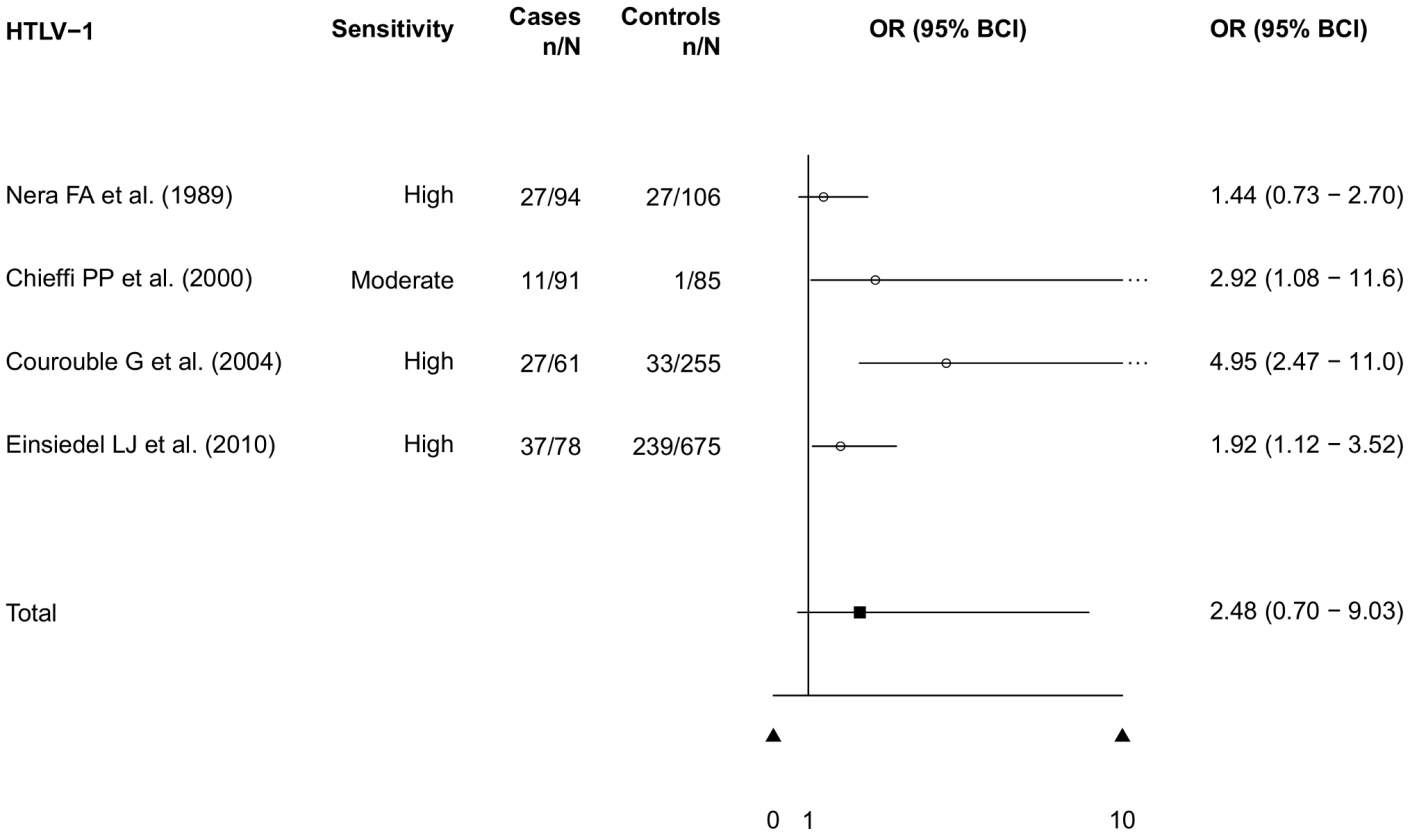
Risk of *S. stercoralis* infection in patients with HTLV-1 infection (meta-analysis of case-control studies).

Persons infected with human T-lymphotropic virus 1 (HTLV-1) tend to be significantly co-infected with *S. stercoralis* in comparison with HTLV-1-seronegative controls [71]–[74]. Our meta-analysis resulted in a pooled OR of 2.48 (95% BCI: 0.70–9.03) for the infection with HTLV-1 [75]–[78] (Figure 8), showing no statistically significant difference. In HTLV-1 infected patients, eradication of the parasite by conventional drug therapy is hindered [79]. *S. stercoralis* hyperinfection syndrome, including its fatal outcome, is particularly common in these patients [80]. *S. stercoralis* co-infection appears to shorten the latency period until the onset of adult T-cell leukaemia in HTLV-1 positive subjects [81].

#### Alcoholics

**Figure 9:**
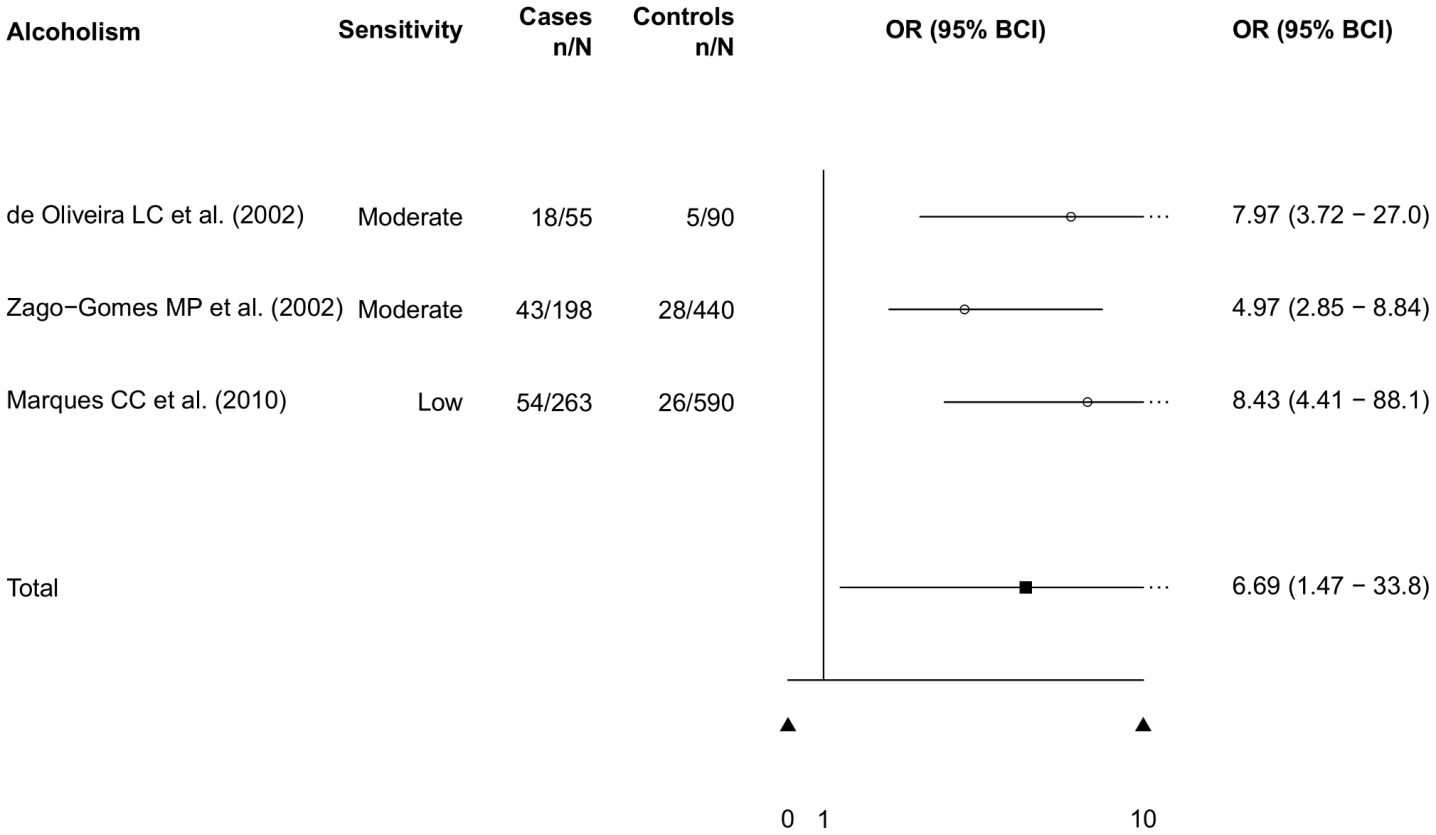
Risk of *S. stercoralis* infection in alcoholics (meta-analysis of case-control studies).

Four studies (three case-control studies, and one cross-sectional study) focused on patients with an alcohol addiction. The case-control studies, all from Brazil, showed higher infection rates in alcoholics than in the control groups [82]–[84]. The meta-analysis resulted in a pooled OR of 6.69 (95% BCI: 1.47–33.8, Figure 9). The study by Zago-Gomes and colleagues [83] showed that only *S. stercoralis* infection rates differed between alcoholics and control groups. Contrastingly, other nematodes showed the same prevalence in alcoholics and control groups. Zago-Gomes and colleagues argue that alcoholics' regular ethanol intake might lead to an immune modulation and/or alteration in corticosteroid metabolism, favoring *S. stercoralis* infection.

#### Patients with diarrhea

**Figure 10:**
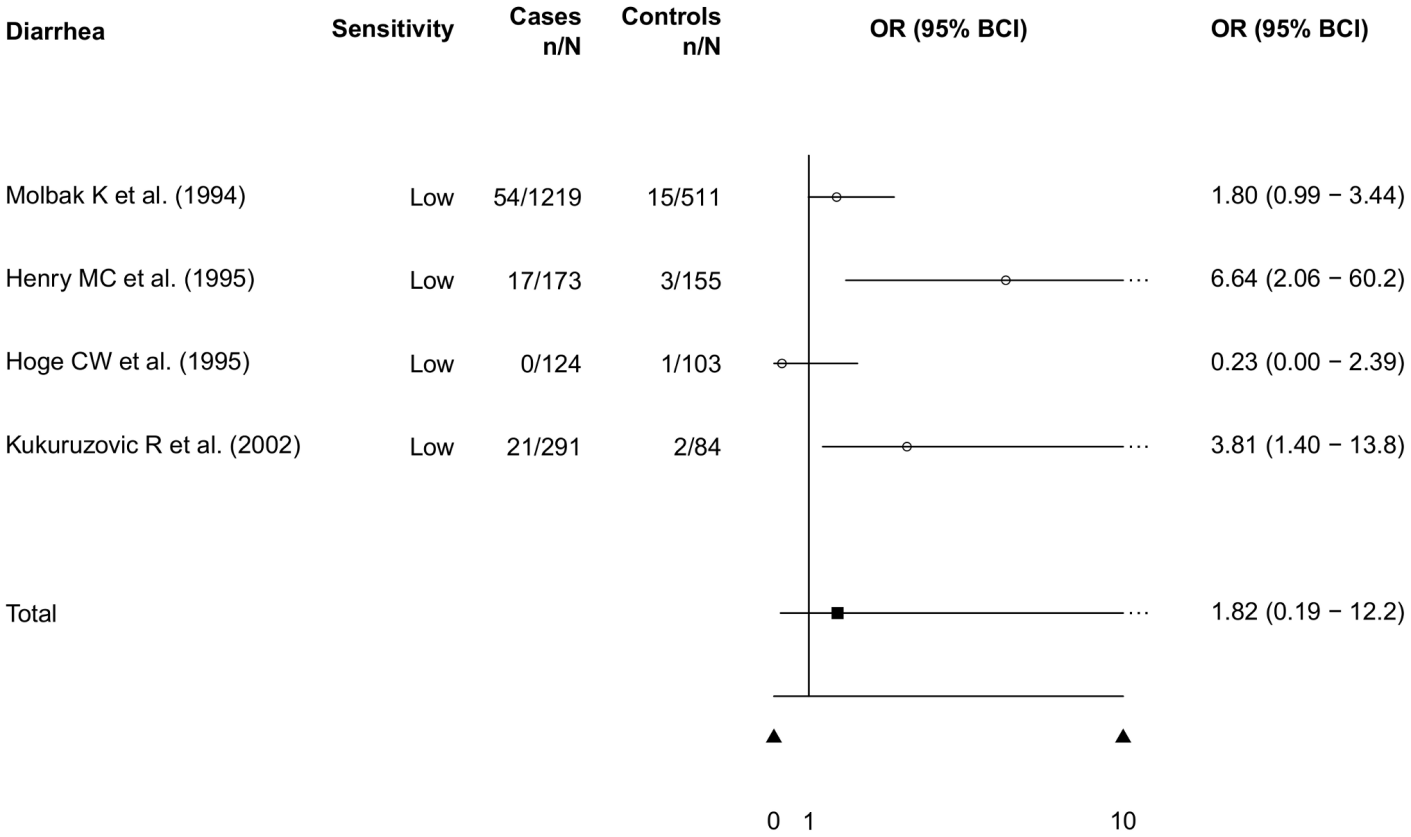
Risk of *S. stercoralis* infection in patients with diarrhea (meta-analysis of case-control studies).

Studies undertaken in patients with diarrhea showed a wide range of infection prevalences. The lowest infection rate was 1.0% (95% CI: 0.0–3.0%) found in a tertiary care hospital in Andhra Pradesh in India [85], while the highest reported was 76.0% (95% CI: 39.0–99.0%) in a study on Cambodian children in a refugee camp at the Thai-Cambodian border [86]. Comparing case-control studies lead to a pooled OR of 1.82 (95% BCI 0.19–12.2), showing no statistically significant difference [87]–[90]. Case-control studies on patients with and without diarrhea are relatively scarce, especially studies reporting on *S. stercoralis*, of which we could only identify four. Because diarrhea is one of the symptoms associated with *S. stercoralis* infection, as well as with other STH-infections, it remains unclear whether diarrhea can be considered as a risk factor, or if infection with STHs leads to a higher prevalence of diarrhea (Figure 10).

#### Patients with malignancies and/or immuno-compromising conditions

Case-control studies often focus on the infestation rates among patients with haematologic neoplastic diseases and/or immuno-suppressing conditions, arising, for instance, as a consequence of treatment. Two studies from Egypt show that *S. stercoralis* is found more often in patients with malignant diseases undergoing immuno-suppressive treatment [91], [92]. In Japan, Hirata and colleagues [93] found the parasite more often in patients diagnosed with biliary tract or pancreatic cancer. The infection rate was 7.5% among the 1,458 controls, 18.4% in the biliary tract cancer group, and 15.4% in the pancreatic cancer group. The liver cancer group reported the same infection rate (7.5%) of strongyloidiasis as the control group. One case-control study from Brazil found *S. stercoralis* to be more prevalent in immuno-compromised children in comparison with an immuno-competent control population by using serological techniques only. Four different serological approaches were used, each reporting higher infection rates in immuno-compromised children (e.g. ELISA-IgG: 12.1% versus 1.5%) than in the control group. No differences could be demonstrated (2.4% versus 4.4%) when based on parasitological examinations of stool samples, using the Baermann method, for three consecutive days [94].

The malignancies and immuno-comprising conditions reported in the literature are manifold, leading to a very heterogeneous set of data. This makes meta-analysis virtually impossible.

#### Children

Of the 354 studies, 84 (23.7%) were conducted specifically on children, adolescents and young adults (aged 0–20 years). One third of them 29 (34.5%) were conducted in Africa, followed by 22 (26.2%) in the Americas and 19 (22.6%) in South-East Asia. The Western Pacific region (9), Middle East (4) and Europe (1) make up the remaining 14 (16.7%) studies. Almost all of these studies are cross-sectional and focus on children only.

Seven studies compared children with adults, but their comparison is challenged by very heterogeneous age grouping and matching. Two studies were conducted in Indonesia; Mangali and colleagues [95] reported a prevalence of 4.4% in the group aged 2–14 years, and 6.7% in all participants aged 15 or older. The study by Toma and colleagues [96] reported similar trends with a prevalence of 0% in the group aged 4–14 years and 1.2% in all participants aged 15 years or older. The study by Dancesco and colleagues [97] in Côte d'Ivoire presented a prevalence of 12.2% in children aged 4–15 years, and 17.7% in adults, also underlining the trend of children having lower prevalence rates than adults. In contrast, the study by Gaburri and colleagues [84] showed a prevalence of 1.9% in adults, and 13.2% in children. The Gaburri study, however, focused on hepatic cirrhosis patients, and the prevalence rates are derived from only partially matched control groups. In Nepal, the study by Navitsky and colleagues [98] found a prevalence of 2.0% in 292 pregnant women (aged 15–40 years) and 0% in 129 infants (aged 10–20 weeks). The study by Wongjindanon and colleagues [99] found a prevalence of 9.7% in adult volunteers in Surin (rural), Thailand, while the prevalence in schoolchildren from Samut Sakhon (suburban) was 2.0%. Due to the heterogeneity of the reported data, meta-analysis was not performed.

### Diagnostic test sensitivity estimation

Estimations of the three diagnostic test sensitivity groups (low, moderate and high) are presented in the Appendix (Table A1–A3). Medians and 95% credible intervals are shown under two different prior specifications and divided according to the study type. Estimates were robust to the prior specification, however they varied among the different study types. Hospital-based surveys led to higher sensitivity estimates than the community-based ones. Sensitivity estimates in the low sensitivity group range from 0.15 to 0.18 in the community-based surveys and from 0.17 to 0.21 in the hospital-based surveys. Sensitivity in the moderate sensitivity group is estimated between 0.77 and 0.90 in the community-based surveys. Higher uncertainty is observed in the estimation of the same diagnostic tools in hospital-based surveys, probably due to a smaller sample sizes. Sensitivity estimates in serological tests vary between 0.88 and 0.98 in community-based studies whereas they are more precise in the hospital-based surveys (0.94–0.98). The meta-analysis included limited number of surveys on immigrants and therefore the corresponding sensitivity estimates can not be compared to those from community- or hospital-based surveys.

## Discussion

### Prevalence rates of *S. stercoralis*

World-wide prevalence rates of *S. stercoralis* have been estimated on several occasions. Values vary from three million to one-hundred million infected individuals [2], [21], [100]–[102]. In 1989, after having examined the epidemiological evidence, Genta [2] called these estimates “little more than inspired guesses” and cast doubts on the “practical value” of those numbers. In fact, knowledge on country and regional *S. stercoralis* infection rates and risks in specific population groups is of increasing clinical and epidemiological importance. Infected individuals are at risk of developing complicated strongyloidiasis as soon as cell-mediated immunity is compromised. The widespread and increasing use of corticosteroids for immuno-suppressive treatment, especially in *S. stercoralis* endemic areas, exacerbates the risk for severe complications associated with this infection.

Our findings provide an overview of the global prevalence of *S. stercoralis*, drawn from published infection reports since 1989. For the first time, we report prevalence rates on a country-by-country basis, based on published infection rates and taking into account the sensitivity of the diagnostic methods used. In Africa, the range of infection rates in the communities varies from 0.1% in the Central African Republic to up to 91.8% in Gabon. In South- and Central-America, Haiti reports a prevalence of 1.0%, while in Peru the infection rate is as high as 75.3%. Interestingly, in South-East Asia, another highly endemic part of the world, several countries report infection rates within a comparably small range. In Cambodia, the infection rate is 17.5%, Thailand 23.7% and Lao PDR 26.2%. Only Vietnam, with a prevalence of 0.02% - based on only one study - falls out of this picture.

In general, information on infection rates/prevalence of the parasite is scarce, and the studies we analyzed suggest that infection with *S. stercoralis* is highly underreported, especially in Sub-Saharan Africa and Southeast Asia. The main reason is that almost no studies focusing on *S. stercoralis* were conducted. Therefore, studies reporting *S. stercoralis* prevalence most often used low-sensitivity diagnostic methods for *S. stercoralis* and only samples from one day were analyzed. Furthermore, information about at-risk groups and affected populations is missing, as few studies focus on strongyloidiasis and possible at-risk groups.

*S. stercoralis* has a very low prevalence in societies where fecal contamination of soil is rare. Hence, it is a very rare infection in developed countries and is less prevalent in urban than in rural areas of resource poor countries, with the exception of slum areas in the bigger cities. In Europe and in the United States the infection occurs in pockets and predominantly affects individuals pursuing farming activities or miners. In Germany, *S. stercoralis* is recognized as a parasitic professional disease in miners [103]. Moreover, in developed countries, strongyloidiasis remains an issue for immigrants [33], [104], tourists [51] and military [53] returning from deployment in endemic areas. This fact has implications for medical services in developed countries, and may call for systematic screening after visits to endemic countries and before initiation of immuno-suppressive treatment.

While information on *S. stercoralis* infection rate is patchy, information on incidence is virtually non-existent. None of the identified studies offered evidence on first or new infections. Incidence rates would give insight into how often and how quickly people are re-infected after successful treatment. Further, it could establish how often first-time infections are sustained over a longer period. We showed that prevalence rates in children are often lower than in adults, yet the incidence might be a lot higher if in fact many adult patients acquired the infection during childhood. In addition, risk for infection might be different in children than in adults. Longitudinal studies, particularly at community level, are required to address this knowledge gap.

Comparing the infection rates from hospitalized patients and infection rates in the communities in the same countries often shows great differences. Venezuela and Zambia are good examples, reporting infection rates of 48.4% and 50.6% in hospitalized persons, respectively; yet in the communities the reported infection rates are as low as 2.3% and 6.6%, respectively. One reason for this discrepancy comes from the use of low-sensitivity methods in community-based studies versus use of moderate- and high-sensitivity methods in the hospitals. Furthermore, hospitalized persons are more likely to belong to an at-risk group or have underlying risk factors for infection with *S. stercoralis*. Additionally, in the hospitals, patients are sampled for more than one day. Another factor is the small number of studies contributing to the calculation of the infection rates. For countries with many studies available (most notably Brazil and Thailand), the differences between the infection rates in communities and in hospitals are considerably smaller (Brazil 13.0% vs. 17.0% and Thailand 23.7% vs. 34.7%). These findings imply that countries with few community-level studies that report high infection rates in the hospitals are likely to be highly endemic. Examples might include DR Congo and Madagascar, both of which lack studies undertaken at community-level yet report infection rates of 32.7% and 52.2% in hospitalized persons, respectively. Here, cross-sectional studies at community level that apply high-sensitivity diagnostic methods and that preferably investigate several stool samples per person over consecutive days are desperately needed to identify possible hotspots of *S. stercoralis* transmission and to quantify the infection rates and risks.

With our approach, we can for the first time report country-wide infection rates. Yet, sometimes a large part of the studies were conducted in a comparatively small area in a specific country. This presents a limitation to our analysis, as do countries with only one or a few studies from a specific location, as it is not possible to make a general statement about prevalence that encompasses all parts of the country. It is very likely that the studies were conducted in areas where *S. stercoralis* infection was already suspected. This is especially true for bigger countries that often have a wide variety of ecological and economic environments, different standards of sanitation, and big differences between rural and urban environments.

A major challenge of giving an overview of prevalence data for *S. stercoralis* world-wide lies in the low comparability of the studies reporting infection rates. Most studies that we identified did not focus on *S. stercoralis* specifically, but on other STHs. Therefore, *S. stercoralis* is mostly reported as an additional outcome and the diagnostic methods used possess only a low sensitivity for *S. stercoralis*. Direct smears and the Kato-Katz method were most commonly used, both of which show a very low sensitivity for the diagnosis of *S. stercoralis* [5], [6], [23]. The more sensitive and *Strongyloides* specific methods, such as the Baermann method and Koga Agar plate culture are more cumbersome and/or time- and resource intensive [7]. In our model for estimating country-wide infection rates, we addressed this limitation by taking into account the sensitivity of the diagnostic methods used, summarized as a range derived from the literature.

To further increase diagnostic sensitivity, more than one stool sample should be examined from the same individual over consecutive days [105]–[108]. This is also true for superior methods like Baermann or Koga Agar plate culture [109], [110]. This is necessary because of the irregular excretion pattern of *S. stercoralis* larvae. Especially for low-intensity infections, there is a big risk that a one-day examination will miss the infection altogether. However, in most studies, only one stool sample was examined. Therefore, the reported infection rates are very likely underestimations.

The challenges outlined above lead to a very heterogeneous set of prevalence data. Today, many countries (including some of the most populous ones) with ecologically and socio-economic conditions favorable to *S. stercoralis* transmission are lacking prevalence data entirely. More data is required for almost all countries and for various socio-economic/cultural settings. Further large-scale surveys that sample the general population, and use highly sensitive methods over three consecutive days would help to narrow this gap.

Finally, as comprehensive as the collection of information on global *S. stercoralis* infection rates was, important information might have been missed due to language restrictions and the choice of databases searched.

### Risk groups for *S. stercoralis* infection

Several possible risk factors for *S. stercoralis* infection are reported in the literature. However, studies that focus specifically on risk groups are very rare. We conducted a meta-analysis of case-control studies that provided information on risk and control groups. Most studies were related to HIV/AIDS infection. Our analysis showed an *S. stercoralis* infection risk for HIV/AIDS patients that was twice as high as the risk for individuals without HIV/AIDS (OR: 2.17, 95% BCI: 1.18–4.01). Most studies used the same diagnostic methods for cases and controls, yet the study of Feitosa and colleagues [59] used additional high sensitivity methods in the HIV-positive group. Another significant highly increased risk for *S. stercoralis* infection was alcoholism (OR: 6.69, 95% BCI: 1.47–33.8). The well-established risk factors HTLV-1 infection as well as diarrhea both showed an increased risk, but without statistical significance (OR: 2.48, 95% BCI: 0.70–9.03 and OR: 1.82, 95% BCI: 0.19–12.2, respectively).

Cases for which strongyloidiasis would cause severe complications in HIV-infected persons are rare. As Keiser & Nutman [11] pointed out, less than 30 cases of hyperinfection in HIV-infected individuals have been reported in the literature thus far. The modulation of the immune system by the HIV appears to be the main reason for this. The increase of TH2 cytokines and the decrease of TH1 cytokines [111]–[113] leads to a pattern that may favor bacterial and viral opportunistic infections rather than helminthic infections [9]. Further, it has been proposed that indirect larval development is promoted in patients that are immuno-compromised by advancing AIDS and therefore, the possibility of increased auto-infection is reduced [114].

All case-control studies included in the meta-analysis for HTLV-1 [75]–[78] showed an increased risk for *S. stercoralis* co-infection for individuals with an HTLV-1 infection. The result of the meta-analysis however showed no statistically significant risk increase in HTLV-1 infected individuals. As there were only four studies that could be included in the meta-analysis, which is a possible limitation, further case-control studies would be needed to come to a unifying conclusion.

Alcohol-addiction is another potential risk factor for *S. stercoralis* infection. Studies undertaken in Brazil [82], [83], [115] showed evidence of this. It is argued that the regular ethanol intake modulates immune response, making survival and reproduction of the larvae in the duodenum easier. Consequently, there is a higher frequency of larvae present in the stools of alcoholic patients, yet an increased infection rate is not necessarily observed.

For patients with malignancies and/or immuno-compromising conditions, case-control studies are also scarce. De Paula and colleagues [94] showed a higher prevalence of *S. stercoralis* in immuno-compromised children compared to immuno-competent children, although these differences could only be shown with serological diagnostic methods. Using coprological methods, there was no difference in prevalence found between the two groups. This might be because serological diagnostic methods are known to cross-react with other helminth infections or because of the higher sensitivity. Three other case control studies showed a higher prevalence in patients with malignant diseases or undergoing immuno-supressive treatment [91]–[93].

Age-related findings suggest that children are not generally at a higher risk for *S. stercoralis* infection. However, behavioral factors might increase the risk of infection, and many of the infected adults might have picked up an infection during childhood and sustained it through auto-infection. The infection rates in children lower than or equal to those in adults suggests that due to the persistence of *S. stercoralis*, infections are accumulated over time. Longitudinal studies are needed to get more insight into the incidence and possible accumulation, following the same individuals over longer time periods.

Discerning the risk factors or possible risk factors for *S. stercoralis* infection is hindered by the small amount of research on *S. stercoralis* in general. Therefore, for most risk factors, only a few case-control studies exist, making it difficult to present clear statements. However, these studies can point to trends and lead the way for further and more detailed research.

### Diagnostic test sensitivity

Diagnostic tests with low or moderate sensitivity underestimate disease prevalence. The inclusion of the diagnostic test sensitivity in the models allowed us to properly evaluate prevalence and OR for the risk factors under study. The sensitivity adjusted OR for each risk factor have larger uncertainty (wider BCI) most likely due to the added variability of the detection. Furthermore, the intensity of infection influences the sensitivity estimates [5]. Higher sensitivity estimates in hospital based surveys may reflect high intensity probably due to co-infection. Test-specific diagnostic sensitivity could not be obtained because of the variety of tests employed in the studies reviewed and relatively small sample size for each test.

### What should be done next?

We showed that in many countries, prevalence of *S. stercoralis* infection is high. The results are based on studies that often do not focus on *S. stercoralis* specifically, but on other STHs. Therefore, the results are mostly based on low-sensitivity diagnostic methods and likely underestimate prevalence. It is necessary to conduct further studies using high sensitivity diagnostic methods, coprologically the Koga Agar plate culture or the Baermann or the ELISA in serology, to achieve a more comprehensive and detailed picture of the global prevalence of *S. stercoralis*. Especially in countries with favorable conditions for *S. stercoralis* transmission, studies conducted on STHs should not neglect to include *S. stercoralis*. This would help to establish more detailed data on regional and country-wide prevalence rates. The results obtained in these studies and of our analysis show many countries with a high estimation of the prevalence rate of *S. stercoralis*. In many of these countries the current policy guidelines neglect or are unclear about how to address *S. stercoralis*. We conclude that *S. stercoralis* is of high importance in global helminth control and should therefore not be neglected.

## Supporting Information

Checklist S1 PRISMA Checklist.
(DOCX)

Diagram S1 PRISMA Flow diagram.
(DOCX)

References S1 Web-based reference list.
(DOC)

Text S1 Appendix: Estimation of country-specific prevalence and estimation of prevalence in specific risk groups.
(DOC)
