# Supplementary material for: Strongyloides stercoralis: Global Distribution and Risk Factors
Source: PLoS Negl Trop Dis. 2013 Jul 11;7(7):e2288. doi: 10.1371/journal.pntd.0002288 (PMC3708837; doi:10.1371/journal.pntd.0002288)
Supplement: Diagram S1 — PRISMA Flow diagram. (DOCX) [file pntd.0002288.s002.docx]

**PRISMA 2009 Flow Diagram**


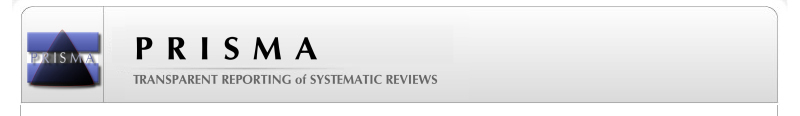


Records excluded (no data on prevalence)
(n = ~1150)

Records identified through database searching
(n = 5274)

Records after duplicates removed (duplicates and non eligible)
(n = ~3770)

Studies included in quantitative prevalence calculation

(n = 354)

(n = )

Studies included in quantitative synthesis (meta-analysis)
(n = 27)

Full-text articles assessed for eligibility
(n = 354)

Records screened
(n = ~1500)

## Identification

## Eligibility

## Included

## Screening
