# Supplementary material for: Strongyloides stercoralis: Global Distribution and Risk Factors
Source: PLoS Negl Trop Dis. 2013 Jul 11;7(7):e2288. doi: 10.1371/journal.pntd.0002288 (PMC3708837; doi:10.1371/journal.pntd.0002288)
Supplement: References S1 — Web-based reference list. (DOC) [file pntd.0002288.s003.doc]

**References**

1. Borda CE, Rea MJ, Rosa JR, Maidana C (1996) Intestinal parasitism in San Cayetano, Corrientes, Argentina. Bull Pan Am Health Organ 30: 227-233.

2. Beltramino D, Lura MC, Carrera E (2003) [Selective vs. mass treatment with antihelminthic drugs: experience in two hyperendemic communities]. Rev Panam Salud Publica 13: 10-18.

3. Taranto NJ, Bonomi de Filippi H, Orione O (1993) [Prevalence of *Strongyloides stercoralis* infection in childhood. Oran, Salta, Argentina]. Bol Chil Parasitol 48: 49-51.

4. Taranto NJ, Cajal SP, De Marzi MC, Fernandez MM, Frank FM, et al. (2003) Clinical status and parasitic infection in a Wichi Aboriginal community in Salta, Argentina. Trans R Soc Trop Med Hyg 97: 554-558.

5. Mendez OC, Szmulewicz G, Menghi C, Torres S, Gonzalez G, et al. (1994) [Comparison of intestinal parasite infestation indexes among HIV positive and negative populations]. Medicina (B Aires) 54: 307-310.

6. Zonta ML, Oyhenart EE, Navone GT (2010) Nutritional status, body composition, and intestinal parasitism among the Mbya-Guarani communities of Misiones, Argentina. Am J Hum Biol 22: 193-200.

7. Krolewiecki AJ, Ramanathan R, Fink V, McAuliffe I, Cajal SP, et al. (2010) Improved diagnosis of *Strongyloides stercoralis* using recombinant antigen-based serologies in a community-wide study in northern Argentina. Clin Vaccine Immunol 17: 1624-1630.

8. Repetto SA, Duran PA, Lasala MB, Gonzalez-Cappa SM (2010) High rate of strongyloidosis infection, out of endemic area, in patients with eosinophilia and without risk of exogenous reinfections. Am J Trop Med Hyg 82: 1088-1093.

9. Ingen IV (2003) Strongyloidiasis in an island community. Progress of a treatment programme. Second National Strongyloidiasis Workshop Brisbane.

10. Oliver NW, Rowbottom DJ, Sexton P, Goldsmid JM, Byard R, et al. (1989) Chronic strongyloidiasis in Tasmanian veterans--clinical diagnosis by the use of a screening index. Aust N Z J Med 19: 458-462.

11. Martin JA, Mak DB (2006) Changing faces: A review of infectious disease screening of refugees by the Migrant Health Unit, Western Australia in 2003 and 2004. Med J Aust 185: 607-610.

12. Rice JE, Skull SA, Pearce C, Mulholland N, Davie G, et al. (2003) Screening for intestinal parasites in recently arrived children from East Africa. J Paediatr Child Health 39: 456-459.

13. Prociv P, Luke R (1993) Observations on strongyloidiasis in Queensland aboriginal communities. Med J Aust 158: 160-163.

14. Meloni BP, Thompson RC, Hopkins RM, Reynoldson JA, Gracey M (1993) The prevalence of *Giardia* and other intestinal parasites in children, dogs and cats from aboriginal communities in the Kimberley. Med J Aust 158: 157-159.

15. Flannery G, White N (1993) Immunological Parameters in Northeast Arnhem-Land Aborigines - Consequences of Changing Settlement-Patterns and Life-Styles. Urban Ecology and Health in the Third World 32: 202-220.

16. Fisher D, McCarry F, Currie B (1993) Strongyloidiasis in the Northern Territory. Under-recognised and under-treated? Med J Aust 159: 88-90.

17. de Silva S, Saykao P, Kelly H, MacIntyre CR, Ryan N, et al. (2002) Chronic *Strongyloides stercoralis* infection in Laotian immigrants and refugees 7-20 years after resettlement in Australia. Epidemiol Infect 128: 439-444.

18. Caruana SR, Kelly HA, Ngeow JY, Ryan NJ, Bennett CM, et al. (2006) Undiagnosed and potentially lethal parasite infections among immigrants and refugees in Australia. J Travel Med 13: 233-239.

19. Kukuruzovic R, Robins-Browne RM, Anstey NM, Brewster DR (2002) Enteric pathogens, intestinal permeability and nitric oxide production in acute gastroenteritis. Pediatr Infect Dis J 21: 730-739.

20. Gibney KB, Mihrshahi S, Torresi J, Marshall C, Leder K, et al. (2009) The profile of health problems in African immigrants attending an infectious disease unit in Melbourne, Australia. Am J Trop Med Hyg 80: 805-811.

21. Einsiedel L, Fernandes L (2008) *Strongyloides stercoralis*: a cause of morbidity and mortality for indigenous people in Central Australia. Intern Med J 38: 697-703.

22. Page WA, Dempsey K, McCarthy JS (2006) Utility of serological follow-up of chronic strongyloidiasis after anthelminthic chemotherapy. Trans R Soc Trop Med Hyg 100: 1056-1062.

23. Aspock H, Hassl A (1990) Parasitic infections in HIV patients in Austria: first results of a long-term study. Zentralbl Bakteriol 272: 540-546.

24. Hall A, Conway DJ, Anwar KS, Rahman ML (1994) *Strongyloides stercoralis* in an urban slum community in Bangladesh: factors independently associated with infection. Trans R Soc Trop Med Hyg 88: 527-530.

25. Aimpun P, Hshieh P (2004) Survey for intestinal parasites in Belize, Central America. Southeast Asian J Trop Med Public Health 35: 506-511.

26. Cancrini G, Bartoloni A, Zaffaroni E, Guglielmetti P, Gamboa H, et al. (1998) Seroprevalence of *Toxocara canis*-IgG antibodies in two rural Bolivian communities. Parassitologia 40: 473-475.

27. Tanner S, Leonard WR, McDade TW, Reyes-Garcia V, Godoy R, et al. (2009) Influence of helminth infections on childhood nutritional status in lowland Bolivia. Am J Hum Biol 21: 651-656.

28. Rossi CL, Partel CD, Teixeira AL, Takahashi EE, De Barros-Mazon S, et al. (1993) Strongyloidiasis in Campinas city region (Brazil). Parasitological and serological data. Trop Geogr Med 45: 189-190.

29. Muck AE, Pires ML, Lammie PJ (2003) Influence of infection with non-filarial helminths on the specificity of serological assays for antifilarial immunoglobulin G4. Trans R Soc Trop Med Hyg 97: 88-90.

30. Chieffi PP, Chiattone CS, Feltrim EN, Alves RC, Paschoalotti MA (2000) Coinfection by *Strongyloides stercoralis* in blood donors infected with human T-cell leukemia/lymphoma virus type 1 in Sao Paulo City, Brazil. Mem Inst Oswaldo Cruz 95: 711-712.

31. Cimerman S, Cimerman B, Lewi DS (1999) Prevalence of intestinal parasitic infections in patients with acquired immunodeficiency syndrome in Brazil. Int J Infect Dis 3: 203-206.

32. Costa-Cruz JM, Cardoso ML, Marques DE (1995) Intestinal parasites in school food handlers in the city of Uberlandia, Minas Gerais, Brazil. Rev Inst Med Trop Sao Paulo 37: 191-196.

33. de Oliveira LC, Ribeiro CT, Mendes Dde M, Oliveira TC, Costa-Cruz JM (2002) Frequency of *Strongyloides stercoralis* infection in alcoholics. Mem Inst Oswaldo Cruz 97: 119-121.

34. de Paula FM, de Castro E, Goncalves-Pires M, Marcal M, Campos DM, et al. (2000) Parasitological and immunological diagnoses of strongyloidiasis in immunocompromised and non-immunocompromised children at Uberlandia, State of Minas Gerais, Brazil. Rev Inst Med Trop Sao Paulo 42: 51-55.

35. Graeff-Teixeira C, Leite CS, Sperhacke CL, Fassina K, Petry SM, et al. (1997) Prospective study of strongyloidosis in patients with hematologic malignancies. Rev Soc Bras Med Trop 30: 355-357.

36. Kobayashi J, Hasegawa H, Forli AA, Nishimura NF, Yamanaka A, et al. (1995) Prevalence of intestinal parasitic infection in five farms in Holambra, Sao Paulo, Brazil. Rev Inst Med Trop Sao Paulo 37: 13-18.

37. Nucci M, Portugal R, Pulcheri W, Spector N, Ferreira SB, et al. (1995) Strongyloidiasis in patients with hematologic malignancies. Clin Infect Dis 21: 675-677.

38. Machado ER, Costa-Cruz JM (1998) *Strongyloides stercoralis* and other enteroparasites in children at Uberlandia city, state of Minas Gerais, Brazil. Mem Inst Oswaldo Cruz 93: 161-164.

39. Zago-Gomes MP, Aikawa KF, Perazzio SF, Goncalves CS, Pereira FE (2002) Prevalence of intestinal nematodes in alcoholic patients. Rev Soc Bras Med Trop 35: 571-574.

40. Silva CV, Ferreira MS, Borges AS, Costa-Cruz JM (2005) Intestinal parasitic infections in HIV/AIDS patients: experience at a teaching hospital in central Brazil. Scand J Infect Dis 37: 211-215.

41. de Rezende CH, Costa-Cruz JM, Gennari-Cardoso ML (1997) [Enteroparasitoses in food handlers of the public schools in Uberlandia (Minas Gerais), Brazil]. Rev Panam Salud Publica 2: 392-397.

42. Dias RM, Mangini AC, Torres DM, Vellosa SA, da Silva MI, et al. (1992) [Occurrence of *Strongyloides stercoralis* in patients with acquired immunodeficiency syndrome (AIDS)]. Rev Inst Med Trop Sao Paulo 34: 15-17.

43. Ferreira MS, Nishioka Sde A, Borges AS, Costa-Cruz JM, Rossin IR, et al. (1999) Strongyloidiasis and infection due to human immunodeficiency virus: 25 cases at a Brazilian teaching hospital, including seven cases of hyperinfection syndrome. Clin Infect Dis 28: 154-155.

44. Dorea RC, Salata E, Padovani CR, dos Anjos GL (1996) Control of parasitic infections among school children in the peri-urban area of Botucatu, Sao Paulo, Brazil. Rev Soc Bras Med Trop 29: 425-430.

45. dos Santos JI, Padilha Filho O (1996) [The low sensitivity of the larval culture method (Harada-Mori) in the diagnosis of strongyloidiasis]. Rev Soc Bras Med Trop 29: 51-52.

46. Gaburri D, Gaburri AK, Hubner E, Lopes MH, Ribeiro AM, et al. (1997) [Intestinal parasitosis and hepatic cirrhosis]. Arq Gastroenterol 34: 7-12.

47. Gennari-Cardoso ML, Costa-Cruz JM, de Castro E, Lima LM, Prudente DV (1996) *Cryptosporidium sp*. in children suffering from acute diarrhea at Uberlandia City, State of Minas Gerais, Brazil. Mem Inst Oswaldo Cruz 91: 551-554.

48. Goncalves JF, Tanabe M, Medeiros Fde P, Goncalves FJ, Aca Ida S, et al. (1990) Parasitological and serological studies on amoebiasis and other intestinal parasitic infections in the rural sector around Recife, northeast Brazil. Rev Inst Med Trop Sao Paulo 32: 428-435.

49. Wuhib T, Silva TM, Newman RD, Garcia LS, Pereira ML, et al. (1994) Cryptosporidial and microsporidial infections in human immunodeficiency virus-infected patients in northeastern Brazil. J Infect Dis 170: 494-497.

50. Moreira-Silva SF, Pereira FE (2000) Intestinal nematodes, *Toxocara* infection, and pyogenic liver abscess in children: a possible association. J Trop Pediatr 46: 167-172.

51. Moura H, Fernandes O, Viola JP, Silva SP, Passos RH, et al. (1989) Enteric parasites and HIV infection: occurrence in AIDS patients in Rio de Janeiro, Brazil. Mem Inst Oswaldo Cruz 84: 527-533.

52. Moura EC, Bragazza LM, Coelho MF, Aun SM (1997) [Prevalence of intestinal parasitosis in schoolchildren]. J Pediatr (Rio J) 73: 406-410.

53. Perez E, Gazin P, Furtado A, Miranda P, Marques NM, et al. (2000) [Intestinal parasite infections and schistosomiasis in a poor urban area, in townships of the sugar cane belt and in villages of the semi-arid area of North-East Brazil]. Sante 10: 127-129.

54. Miranda RA, Xavier FB, Menezes RC (1998) [Intestinal parasitism in a Parakana indigenous community in southwestern Para State, Brazil]. Cad Saude Publica 14: 507-511.

55. Willcox HP, Coura JR (1991) The efficiency of Lutz, Kato-Katz and Baermann-Moraes (adapted) techniques association to the diagnosis of intestinal helminths. Mem Inst Oswaldo Cruz 86: 457-460.

56. Aguiar JI, Goncalves AQ, Sodre FC, Pereira Sdos R, Boia MN, et al. (2007) Intestinal protozoa and helminths among Terena Indians in the State of Mato Grosso do Sul: high prevalence of *Blastocystis hominis*. Rev Soc Bras Med Trop 40: 631-634.

57. Machado ER, Santos DS, Costa-Cruz JM (2008) Enteroparasites and commensals among children in four peripheral districts of Uberlandia, State of Minas Gerais. Rev Soc Bras Med Trop 41: 581-585.

58. Mine JC, Rosa JA (2008) Frequency of *Blastocystis hominis* and other intestinal parasites in stool samples examined at the Parasitology Laboratory of the School of Pharmaceutical Sciences at the Sao Paulo State University, Araraquara. Rev Soc Bras Med Trop 41: 565-569.

59. Dreyer G, Fernandes-Silva E, Alves S, Rocha A, Albuquerque R, et al. (1996) Patterns of detection of *Strongyloides stercoralis* in stool specimens: implications for diagnosis and clinical trials. J Clin Microbiol 34: 2569-2571.

60. Feitosa G, Bandeira AC, Sampaio DP, Badaro R, Brites C (2001) High prevalence of *giardia*sis and stronglyloidiasis among HIV-infected patients in Bahia, Brazil. Braz J Infect Dis 5: 339-344.

61. Kobayashi J, Hasegawa H, Soares EC, Toma H, Dacal AR, et al. (1996) Studies on prevalence of *Strongyloides* infection in Holambra and Maceio, Brazil, by the agar plate faecal culture method. Rev Inst Med Trop Sao Paulo 38: 279-284.

62. Marchi Blatt J, Cantos GA (2003) Evaluation of techniques for the diagnosis of *Strongyloides stercoralis* in human immunodeficiency virus (HIV) positive and HIV negative individuals in the city of Itajai, Brazil. Braz J Infect Dis 7: 402-408.

63. Sato Y, Kobayashi J, Toma H, Shiroma Y (1995) Efficacy of stool examination for detection of *Strongyloides* infection. Am J Trop Med Hyg 53: 248-250.

64. Schaffel R, Nucci M, Carvalho E, Braga M, Almeida L, et al. (2001) The value of an immunoenzymatic test (enzyme-linked immunosorbent assay) for the diagnosis of strongyloidiasis in patients immunosuppressed by hematologic malignancies. Am J Trop Med Hyg 65: 346-350.

65. Heukelbach J, Winter B, Wilcke T, Muehlen M, Albrecht S, et al. (2004) Selective mass treatment with ivermectin to control intestinal helminthiases and parasitic skin diseases in a severely affected population. Bull World Health Organ 82: 563-571.

66. Heukelbach J, Wilcke T, Winter B, Sales de Oliveira FA, Saboia Moura RC, et al. (2004) Efficacy of ivermectin in a patient population concomitantly infected with intestinal helminths and ectoparasites. Arzneimittelforschung 54: 416-421.

67. Costa-Cruz JM, Machado ER, Campos DM (1998) Seroepidemiological study of human strongyloidiasis with blood samples collected on filter paper, in Abadia dos Dourados (Minas Gerais, Brazil). Rev Inst Med Trop Sao Paulo 40: 329-331.

68. Ines Ede J, Souza JN, Santos RC, Souza ES, Santos FL, et al. (2011) Efficacy of parasitological methods for the diagnosis of *Strongyloides stercoralis* and hookworm in faecal specimens. Acta Trop 120: 206-210.

69. Santos SA, Merlini LS (2010) [Prevalence of enteroparasitosis in the population of Maria Helena, Parana State]. Cien Saude Colet 15: 899-905.

70. Marques CC, da Penha Zago-Gomes M, Goncalves CS, Pereira FE (2010) Alcoholism and *Strongyloides stercoralis*: daily ethanol ingestion has a positive correlation with the frequency of *Strongyloides* larvae in the stools. PLoS Negl Trop Dis 4: e717.

71. Floch PJ, Laroche R, Kadende P, Nkurunziza T, Mpfizi B (1989) [Parasites, etiologic agents of diarrhea in AIDS. Significance of duodenal aspiration fluid test]. Bull Soc Pathol Exot Filiales 82: 316-320.

72. Chhakda T, Muth S, Socheat D, Odermatt P (2006) Intestinal parasites in school-aged children in villages bordering Tonle Sap Lake, Cambodia. Southeast Asian J Trop Med Public Health 37: 859-864.

73. Longfils P, Heang UK, Soeng H, Sinuon M (2005) Weekly iron and folic acid supplementation as a tool to reduce anemia among primary school children in Cambodia. Nutr Rev 63: S139-145.

74. Koga-Kita K (2004) Intestinal parasitic infections and socioeconomic status in Prek Russey Commune, Cambodia. Nihon Koshu Eisei Zasshi 51: 986-992.

75. Copelovitch L, Sam Ol O, Taraquinio S, Chanpheaktra N (2010) Childhood nephrotic syndrome in Cambodia: an association with gastrointestinal parasites. J Pediatr 156: 76-81.

76. Moyou-Somo R, Kefie-Arrey C, Dreyfuss G, Dumas M (2003) An epidemiological study of pleuropulmonary paragonimiasis among pupils in the peri-urban zone of Kumba town, Meme Division, Cameroon. BMC Public Health 3: 40.

77. Gyorkos TW, Genta RM, Viens P, MacLean JD (1990) Seroepidemiology of *Strongyloides* infection in the Southeast Asian refugee population in Canada. Am J Epidemiol 132: 257-264.

78. Gyorkos TW, MacLean JD, Viens P, Chheang C, Kokoskin-Nelson E (1992) Intestinal parasite infection in the Kampuchean refugee population 6 years after resettlement in Canada. J Infect Dis 166: 413-417.

79. Gyorkos TW, Frappier-Davignon L, MacLean JD, Viens P (1989) Effect of screening and treatment on imported intestinal parasite infections: results from a randomized, controlled trial. Am J Epidemiol 129: 753-761.

80. Germani Y, Minssart P, Vohito M, Yassibanda S, Glaziou P, et al. (1998) Etiologies of acute, persistent, and dysenteric diarrheas in adults in Bangui, Central African Republic, in relation to human immunodeficiency virus serostatus. Am J Trop Med Hyg 59: 1008-1014.

81. Peng HW, Chao HL, Fan PC (1993) Imported *Opisthorchis viverrini* and parasite infections from Thai labourers in Taiwan. J Helminthol 67: 102-106.

82. Cheng HS, Shieh YH (2000) Investigation on subclinical aspects related to intestinal parasitic infections among Thai laborers in Taipei. J Travel Med 7: 319-324.

83. Wang LC (1998) Parasitic infections among Southeast Asian labourers in Taiwan: a long-term study. Epidemiol Infect 120: 81-86.

84. Steinmann P, Zhou XN, Du ZW, Jiang JY, Wang LB, et al. (2007) Occurrence of *Strongyloides stercoralis* in Yunnan Province, China, and comparison of diagnostic methods. PLoS Negl Trop Dis 1: e75.

85. Ordonez LE, Angulo ES (2004) [Efficacy of ivermectin in the treatment of children parasitized by *Strongyloides stercoralis*]. Biomedica 24: 33-41.

86. Botero JH, Castano A, Montoya MN, Ocampo NE, Hurtado MI, et al. (2003) A preliminary study of the prevalence of intestinal parasites in immunocompromised patients with and without gastrointestinal manifestations. Rev Inst Med Trop Sao Paulo 45: 197-200.

87. Hernandez-Chavarria F, Avendano L (2001) A simple modification of the Baermann method for diagnosis of strongyloidiasis. Mem Inst Oswaldo Cruz 96: 805-807.

88. Menan EI, Nebavi NG, Adjetey TA, Assavo NN, Kiki-Barro PC, et al. (1997) [Profile of intestinal helminthiases in school aged children in the city of Abidjan]. Bull Soc Pathol Exot 90: 51-54.

89. Dancesco P, Abeu J, Akakpo C, Iamandi I, Kacou E, et al. (2005) [Intestinal parasitoses in a village of Cote d'Ivoire. I: Control and prevention plan]. Sante 15: 5-10.

90. Therizol-Ferly PM, Tagliante-Saracino J, Kone M, Konan A, Ouhon J, et al. (1989) [Chronic diarrhea and parasitoses in adults suspected of AIDS in the Ivory Coast]. Bull Soc Pathol Exot Filiales 82: 690-693.

91. Becker SL, Sieto B, Silue KD, Adjossan L, Kone S, et al. (2011) Diagnosis, clinical features, and self-reported morbidity of *Strongyloides stercoralis* and hookworm infection in a Co-endemic setting. PLoS Negl Trop Dis 5: e1292.

92. Glinz D, N'Guessan NA, Utzinger J, N'Goran EK (2010) High prevalence of *Strongyloides stercoralis* among school children in rural Cote d'Ivoire. J Parasitol 96: 431-433.

93. Henry MC, Alary M, Desmet P, Gerniers M, Muteteke D, et al. (1995) Community survey of diarrhoea in children under 5 years in Kinshasa, Zaire. Ann Soc Belg Med Trop 75: 105-114.

94. Adedayo O, Grell G, Bellot P (2003) Hospital admissions for human T-cell lymphotropic virus type-1 (HTLV-1) associated diseases in Dominica. Postgrad Med J 79: 341-344.

95. Cooper PJ, Guevara A, Guderian RH (1993) Intestinal helminthiases in Ecuador: the relationship between prevalence, genetic, and socioeconomic factors. Rev Soc Bras Med Trop 26: 175-180.

96. Jacobsen KH, Ribeiro PS, Quist BK, Rydbeck BV (2007) Prevalence of intestinal parasites in young Quichua children in the highlands of rural Ecuador. J Health Popul Nutr 25: 399-405.

97. Moustafa MA (1997) An evaluation of the modified agar plate method for diagnosis of *Strongyloides stercoralis*. J Egypt Soc Parasitol 27: 571-579.

98. Salem SA, Mohamed NH, Azab ME, Soffa SA, el Kadery AA, et al. (1990) A survey for enteroparasites in Menoufia governorate, Egypt with special reference to *Strongyloides stercoralis*. J Egypt Soc Parasitol 20: 335-344.

99. Mohamed NH, Salem SA, Azab ME, Bebars MA, Khattab HM, et al. (1991) Parasitic infections associated with mental retardation in Egypt. J Egypt Soc Parasitol 21: 319-331.

100. Abaza SM, Makhlouf LM, el-Shewy KA, el-Moamly AA (1995) Intestinal opportunistic parasites among different groups of immunocompromised hosts. J Egypt Soc Parasitol 25: 713-727.

101. el-Sahly AM, Zakaria S, Ahmed L, Mabrouk MA, Thakeb F, et al. (1990) Intestinal helminthic and protozoal infections and urinary schistosomiasis in Egyptian children. J Egypt Soc Parasitol 20: 9-21.

102. Sadek Y, el-Fakahany AF, Lashin AH, el-Salam FA (1997) Intestinal parasites among food-handlers in Qualyobia Governorate, with reference to the pathogenic parasite *blastocystis hominis*. J Egypt Soc Parasitol 27: 471-478.

103. Khalil HM, Makled MK, Azab ME, Abdalla HM, el Sherif EA, et al. (1991) Opportunistic parasitic infections in immunocompromised hosts. J Egypt Soc Parasitol 21: 657-668.

104. El Shazly AM, Awad SE, Sultan DM, Sadek GS, Khalil HH, et al. (2006) Intestinal parasites in Dakahlia governorate, with different techniques in diagnosing protozoa. J Egypt Soc Parasitol 36: 1023-1034.

105. el-Shazly AM, el-Nahas HA, Soliman M, Sultan DM, Abedl Tawab AH, et al. (2006) The reflection of control programs of parasitic diseases upon gastrointestinal helminthiasis in Dakahlia Governorate, Egypt. J Egypt Soc Parasitol 36: 467-480.

106. El-Sherbini GT, Aboul Noor MF, Hegazi MM (2008) Parasitiosis in handicapped children in an Egyptian blind asylum. J Egypt Soc Parasitol 38: 319-326.

107. Mousa KM, Abdel-Tawab AH, Khalil HH, El-Hussieny NA (2010) Diarrhea due to parasites particularly *Cryptosporidium parvum* in great Cairo, Egypt. J Egypt Soc Parasitol 40: 439-450.

108. Azab ME, Mohamed NH, Salem SA, Safar EH, Bebars MA, et al. (1992) Parasitic infections associated with malignancy and leprosy. J Egypt Soc Parasitol 22: 59-70.

109. Assefa T, Woldemichael T, Seyoum T (1991) Evaluation of the modified Baermann's method in the laboratory diagnosis of *Strongyloides stercoralis*. Ethiop Med J 29: 193-198.

110. Fontanet AL, Sahlu T, Rinke de Wit T, Messele T, Masho W, et al. (2000) Epidemiology of infections with intestinal parasites and human immunodeficiency virus (HIV) among sugar-estate residents in Ethiopia. Ann Trop Med Parasitol 94: 269-278.

111. Birrie H, Erko B, Tedla S (1994) Intestinal helminthic infections in the southern Rift Valley of Ethiopia with special reference to schistosomiasis. East Afr Med J 71: 447-452.

112. Lo CT, Ayele T, Birrie H (1989) Helminth and snail survey in Harerge region of Ethiopia with special reference to schistosomiasis. Ethiop Med J 27: 73-83.

113. Mengesha B (1994) Cryptosporidiosis among medical patients with the acquired immunodeficiency syndrome in Tikur Anbessa Teaching Hospital, Ethiopia. East Afr Med J 71: 376-378.

114. Tadesse A, Kassu A (2005) Intestinal parasite isolates in AIDS patients with chronic diarrhea in Gondar Teaching Hospital, North west Ethiopia. Ethiop Med J 43: 93-96.

115. Wondimagegnehu T, Woldemichael T, Assefa T (1992) Hookworm infection among the Melka Sedi banana plantation residents, middle Awash Valley, Ethiopia. Ethiop Med J 30: 129-134.

116. Hailemariam G, Kassu A, Abebe G, Abate E, Damte D, et al. (2004) Intestinal parasitic infections in HIV/AIDS and HIV seronegative individuals in a teaching hospital, Ethiopia. Jpn J Infect Dis 57: 41-43.

117. Endeshaw T, Mohammed H, Woldemichael T (2004) *Cryptosporidium parvum* and other instestinal parasites among diarrhoeal patients referred to EHNRI in Ethiopia. Ethiop Med J 42: 195-198.

118. Worku N, Erko B, Torben W, Belay M, Kasssu A, et al. (2009) Malnutrition and intestinal parasitic infections in school children of Gondar, North West Ethiopia. Ethiop Med J 47: 9-16.

119. Huruy K, Kassu A, Mulu A, Worku N, Fetene T, et al. (2011) Intestinal parasitosis and shigellosis among diarrheal patients in Gondar teaching hospital, northwest Ethiopia. BMC Res Notes 4: 472.

120. Getaneh A, Medhin G, Shimelis T (2010) *Cryptosporidium* and *Strongyloides stercoralis* infections among people with and without HIV infection and efficiency of diagnostic methods for *Strongyloides* in Yirgalem Hospital, southern Ethiopia. BMC Res Notes 3: 90.

121. Thomas M, Woodfield G, Moses C, Amos G (2005) Soil-transmitted helminth infection, skin infection, anaemia, and growth retardation in schoolchildren of Taveuni Island, Fiji. N Z Med J 118: U1492.

122. Molinier S, Chaudier B, Kraemer P, Graffin B, San VV, et al. (1998) [Diagnostic and treatment of hypereosinophilia upon return from the tropics: 102 patients]. Med Trop (Mars) 58: 499-502.

123. Lamour P, Bouree P, Hennequin C, Lombrail P, Squinazi F, et al. (1994) [Blind treatment or treatment oriented to intestinal parasitoses in a Parisian health center for refugees]. Sante 4: 21-26.

124. Gendrel D, Richard-Lenoble D, Kombila M, Nardou M, Gahouma D, et al. (1992) [Decreased intraepithelial lymphocytes in the intestinal mucosa in children with malnutrition and parasitic infections]. Ann Pediatr (Paris) 39: 95-98.

125. Yelifari L, Bloch P, Magnussen P, van Lieshout L, Dery G, et al. (2005) Distribution of human *Oesophagostomum bifurcum*, hookworm and *Strongyloides stercoralis* infections in northern Ghana. Trans R Soc Trop Med Hyg 99: 32-38.

126. Adjei A, Lartey M, Adiku TK, Rodrigues O, Renner L, et al. (2003) *Cryptosporidium* oocysts in Ghanaian AIDS patients with diarrhoea. East Afr Med J 80: 369-372.

127. Kelley PW, Takafuji ET, Wiener H, Milhous W, Miller R, et al. (1989) An outbreak of hookworm infection associated with military operations in Grenada. Mil Med 154: 55-59.

128. Courouble G, Rouet F, Herrmann-Storck C, Nicolas M, Candolfi E, et al. (2004) Epidemiologic study of the association between human T-cell lymphotropic virus type 1 and *Strongyloides stercoralis* infection in female blood donors (Guadeloupe, French West Indies). West Indian Med J 53: 3-6.

129. Nicolas M, Perez JM, Carme B (2006) [Intestinal parasitosis in French West Indies: endemic evolution from 1991 to 2003 in the University Hospital of Pointe-a-Pitre, Guadeloupe]. Bull Soc Pathol Exot 99: 254-257.

130. Villar J, Klebanoff M, Kestler E (1989) The effect on fetal growth of protozoan and helminthic infection during pregnancy. Obstet Gynecol 74: 915-920.

131. Gyorkos TW, Camara B, Kokoskin E, Carabin H, Prouty R (1996) [Survey of parasitic prevalence in school-aged children in Guinea (1995)]. Sante 6: 377-381.

132. Glickman LT, Camara AO, Glickman NW, McCabe GP (1999) Nematode intestinal parasites of children in rural Guinea, Africa: prevalence and relationship to geophagia. Int J Epidemiol 28: 169-174.

133. Lebbad M, Norrgren H, Naucler A, Dias F, Andersson S, et al. (2001) Intestinal parasites in HIV-2 associated AIDS cases with chronic diarrhoea in Guinea-Bissau. Acta Trop 80: 45-49.

134. Molbak K, Wested N, Hojlyng N, Scheutz F, Gottschau A, et al. (1994) The etiology of early childhood diarrhea: a community study from Guinea-Bissau. J Infect Dis 169: 581-587.

135. Champetier de Ribes G, Fline M, Desormeaux AM, Eyma E, Montagut P, et al. (2005) [Intestinal helminthiasis in school children in Haiti in 2002]. Bull Soc Pathol Exot 98: 127-132.

136. de Kaminsky RG (1993) Evaluation of three methods for laboratory diagnosis of *Strongyloides stercoralis* infection. J Parasitol 79: 277-280.

137. Kaminsky RG (1991) Parasitism and diarrhoea in children from two rural communities and marginal barrio in Honduras. Trans R Soc Trop Med Hyg 85: 70-73.

138. Kaminsky RG, Soto RJ, Campa A, Baum MK (2004) Intestinal parasitic infections and eosinophilia in an human immunedeficiency virus positive population in Honduras. Mem Inst Oswaldo Cruz 99: 773-778.

139. Lindo JF, Dubon JM, Ager AL, de Gourville EM, Solo-Gabriele H, et al. (1998) Intestinal parasitic infections in human immunodeficiency virus (HIV)-positive and HIV-negative individuals in San Pedro Sula, Honduras. Am J Trop Med Hyg 58: 431-435.

140. Lanjewar DN, Rodrigues C, Saple DG, Hira SK, DuPont HL (1996) *Cryptosporidium*, *isospora* and *strongyloides* in AIDS. Natl Med J India 9: 17-19.

141. Singh HL, Singh NB, Singh YI (2004) Helminthic infestation of the primary school-going children in Manipur. J Commun Dis 36: 111-116.

142. Singh S, Samantaray JC, Singh N, Das GB, Verma IC (1993) *Trichuris vulpis* infection in an Indian tribal population. J Parasitol 79: 457-458.

143. Kang G, Mathew MS, Rajan DP, Daniel JD, Mathan MM, et al. (1998) Prevalence of intestinal parasites in rural Southern Indians. Trop Med Int Health 3: 70-75.

144. Joshi M, Chowdhary AS, Dalal PJ, Maniar JK (2002) Parasitic diarrhoea in patients with AIDS. Natl Med J India 15: 72-74.

145. Prasad KN, Nag VL, Dhole TN, Ayyagari A (2000) Identification of enteric pathogens in HIV-positive patients with diarrhoea in northern India. J Health Popul Nutr 18: 23-26.

146. Rudrapatna JS, Kumar V, Sridhar H (1997) Intestinal parasitic infections in patients with malignancy. J Diarrhoeal Dis Res 15: 71-74.

147. Subbannayya K, Babu MH, Kumar A, Rao TS, Shivananda PG (1989) *Entamoeba histolytica* and other parasitic infections in south Kanara district, Karnataka. J Commun Dis 21: 207-213.

148. Ananthasubramanian M, Ananthan S, Vennila R, Bhanu S (1997) *Cryptosporidium* in AIDS patients in south India: a laboratory investigation. J Commun Dis 29: 29-33.

149. Nagamani K, Rajkumari A (2001) Cryptosporidiosis in a tertiary care hospital in Andhra Pradesh. Indian J Med Microbiol 19: 215-216.

150. Goka AK, Rolston DD, Mathan VI, Farthing MJ (1990) Diagnosis of *Strongyloides* and hookworm infections: comparison of faecal and duodenal fluid microscopy. Trans R Soc Trop Med Hyg 84: 829-831.

151. Fernandez MC, Verghese S, Bhuvaneswari R, Elizabeth SJ, Mathew T, et al. (2002) A comparative study of the intestinal parasites prevalent among children living in rural and urban settings in and around Chennai. J Commun Dis 34: 35-39.

152. Banerjee D, Deb R, Dar L, Mirdha BR, Pati SK, et al. (2009) High frequency of parasitic and viral stool pathogens in patients with active ulcerative colitis: report from a tropical country. Scand J Gastroenterol 44: 325-331.

153. Vignesh R, Balakrishnan P, Shankar EM, Murugavel KG, Hanas S, et al. (2007) High proportion of isosporiasis among HIV-infected patients with diarrhea in southern India. Am J Trop Med Hyg 77: 823-824.

154. Bangs MJ, Purnomo, Andersen EM, Anthony RL (1996) Intestinal parasites of humans in a highland community of Irian Jaya, Indonesia. Ann Trop Med Parasitol 90: 49-53.

155. Widjana DP, Sutisna P (2000) Prevalence of soil-transmitted helminth infections in the rural population of Bali, Indonesia. Southeast Asian J Trop Med Public Health 31: 454-459.

156. Hasegawa H, Miyagi I, Toma T, Kamimura K, Nainggolan IJ, et al. (1992) Intestinal parasitic infections in Likupang, North Sulawesi, Indonesia. Southeast Asian J Trop Med Public Health 23: 219-227.

157. Mangali A, Sasabone P, Syafruddin, Abadi K, Hasegawa H, et al. (1994) Prevalence of intestinal helminthic infections in Kao District, north Halmahera, Indonesia. Southeast Asian J Trop Med Public Health 25: 737-744.

158. Mangali A, Sasabone P, Syafruddin, Abadi K, Hasegawa H, et al. (1993) Intestinal parasitic infections in Campalagian district, south Sulawesi, Indonesia. Southeast Asian J Trop Med Public Health 24: 313-320.

159. Toma A, Miyagi I, Kamimura K, Tokuyama Y, Hasegawa H, et al. (1999) Questionnaire survey and prevalence of intestinal helminthic infections in Barru, Sulawesi, Indonesia. Southeast Asian J Trop Med Public Health 30: 68-77.

160. Zali MR, Mehr AJ, Rezaian M, Meamar AR, Vaziri S, et al. (2004) Prevalence of intestinal parasitic pathogens among HIV-positive individuals in Iran. Jpn J Infect Dis 57: 268-270.

161. Meamar AR, Rezaian M, Mohraz M, Hadighi R, Kia EB (2007) *Strongyloides stercoralis* hyper-infection syndrome in HIV+/AIDS patients in Iran. Parasitol Res 101: 663-665.

162. Nasiri V, Esmailnia K, Karim G, Nasir M, Akhavan O (2009) Intestinal parasitic infections among inhabitants of Karaj City, Tehran province, Iran in 2006-2008. Korean J Parasitol 47: 265-268.

163. Mahdi NK, Setrak SK, Shiwaish SM (1993) Diagnostic methods for intestinal parasites in southern Iraq with reference to *Strongyloides stercoralis*. Southeast Asian J Trop Med Public Health 24: 685-691.

164. Nahmias J, Greenberg Z, Djerrasi L, Giladi L (1991) Mass treatment of intestinal parasites among Ethiopian immigrants. Isr J Med Sci 27: 278-283.

165. Berger SA, Schwartz T, Michaeli D (1989) Infectious disease among Ethiopian immigrants in Israel. Arch Intern Med 149: 117-119.

166. Huminer D, Symon K, Groskopf I, Pietrushka D, Kremer I, et al. (1992) Seroepidemiologic study of toxocariasis and strongyloidiasis in institutionalized mentally retarded adults. Am J Trop Med Hyg 46: 278-281.

167. Gatti S, Lopes R, Cevini C, Ijaoba B, Bruno A, et al. (2000) Intestinal parasitic infections in an institution for the mentally retarded. Ann Trop Med Parasitol 94: 453-460.

168. Pirisi M, Salvador E, Bisoffi Z, Gobbo M, Smirne C, et al. (2006) Unsuspected strongyloidiasis in hospitalised elderly patients with and without eosinophilia. Clin Microbiol Infect 12: 787-792.

169. Gualdieri L, Rinaldi L, Petrullo L, Morgoglione ME, Maurelli MP, et al. (2011) Intestinal parasites in immigrants in the city of Naples (southern Italy). Acta Trop 117: 196-201.

170. Bisoffi Z, Buonfrate D, Angheben A, Boscolo M, Anselmi M, et al. (2011) Randomized clinical trial on ivermectin versus thiabendazole for the treatment of strongyloidiasis. PLoS Negl Trop Dis 5: e1254.

171. Masucci L, Graffeo R, Bani S, Bugli F, Boccia S, et al. (2011) Intestinal parasites isolated in a large teaching hospital, Italy, 1 May 2006 to 31 December 2008. Euro Surveill 16.

172. Lindo JF, Robinson RD, Terry SI, Vogel P, Gam AA, et al. (1995) Age-prevalence and household clustering of *Strongyloides stercoralis* infection in Jamaica. Parasitology 110 ( Pt 1): 97-102.

173. Rawlins SC, Campbell M, Fox K, Bennett F, Gibbs WN, et al. (1991) Parasitic infections in young Jamaicans in different ecological zones of the island. Trop Geogr Med 43: 136-141.

174. Nera FA, Murphy EL, Gam A, Hanchard B, Figueroa JP, et al. (1989) Antibodies to *Strongyloides stercoralis* in healthy Jamaican carriers of HTLV-1. N Engl J Med 320: 252-253.

175. Arakaki T, Asato R, Ikeshiro T, Sakiyama K, Iwanaga M (1992) Is the prevalence of HTLV-1 infection higher in *Strongyloides* carriers than in non-carriers? Trop Med Parasitol 43: 199-200.

176. Arakaki T, Kohakura M, Asato R, Ikeshiro T, Nakamura S, et al. (1992) Epidemiological aspects of *Strongyloides stercoralis* infection in Okinawa, Japan. J Trop Med Hyg 95: 210-213.

177. Aoyama H, Hirata T, Sakugawa H, Watanabe T, Miyagi S, et al. (2007) An inverse relationship between autoimmune liver diseases and *Strongyloides stercoralis* infection. Am J Trop Med Hyg 76: 972-976.

178. Arakaki T, Iwanaga M, Asato R, Ikeshiro T (1992) Age-related prevalence of *Strongyloides stercoralis* infection in Okinawa, Japan. Trop Geogr Med 44: 299-303.

179. Hirata T, Uchima N, Kishimoto K, Zaha O, Kinjo N, et al. (2006) Impairment of host immune response against *strongyloides stercoralis* by human T cell lymphotropic virus type 1 infection. Am J Trop Med Hyg 74: 246-249.

180. Hirata T, Kishimoto K, Kinjo N, Hokama A, Kinjo F, et al. (2007) Association between *Strongyloides stercoralis* infection and biliary tract cancer. Parasitol Res 101: 1345-1348.

181. Hayashi J, Kishihara Y, Yoshimura E, Furusyo N, Yamaji K, et al. (1997) Correlation between human T cell lymphotropic virus type-1 and *Strongyloides stercoralis* infections and serum immunoglobulin E responses in residents of Okinawa, Japan. Am J Trop Med Hyg 56: 71-75.

182. Arakaki T, Iwanaga M, Kinjo F, Saito A, Asato R, et al. (1990) Efficacy of agar-plate culture in detection of *Strongyloides stercoralis* infection. J Parasitol 76: 425-428.

183. Sato Y, Toma H, Takara M, Shiroma Y (1990) Application of enzyme-linked immunosorbent assay for mass examination of strongyloidiasis in Okinawa, Japan. Int J Parasitol 20: 1025-1029.

184. Sato Y, Toma H, Kiyuna S, Shiroma Y (1991) Gelatin particle indirect agglutination test for mass examination for strongyloidiasis. Trans R Soc Trop Med Hyg 85: 515-518.

185. Toma H, Shimabukuro I, Kobayashi J, Tasaki T, Takara M, et al. (2000) Community control studies on *Strongyloides* infection in a model island of Okinawa, Japan. Southeast Asian J Trop Med Public Health 31: 383-387.

186. Hirata T, Nakamura H, Kinjo N, Hokama A, Kinjo F, et al. (2007) Prevalence of *Blastocystis hominis* and *Strongyloides stercoralis* infection in Okinawa, Japan. Parasitol Res 101: 1717-1719.

187. Ali-Shtayeh MS, Hamdan AH, Shaheen SF, Abu-Zeid I, Faidy YR (1989) Prevalence and seasonal fluctuations of intestinal parasitic infections in the Nablus area, West Bank of Jordan. Ann Trop Med Parasitol 83: 67-72.

188. Ashford RW, Craig PS, Oppenheimer SJ (1992) Polyparasitism on the Kenya coast. 1. Prevalence, and association between parasitic infections. Ann Trop Med Parasitol 86: 671-679.

189. Joyce T, McGuigan KG, Elmore-Meegan M, Conroy RM (1996) Prevalence of enteropathogens in stools of rural Maasai children under five years of age in the Maasailand region of the Kenyan Rift Valley. East Afr Med J 73: 59-62.

190. Walson JL, Stewart BT, Sangare L, Mbogo LW, Otieno PA, et al. (2010) Prevalence and correlates of helminth co-infection in Kenyan HIV-1 infected adults. PLoS Negl Trop Dis 4: e644.

191. Kagira JM, Maina N, Njenga J, Karanja SM, Karori SM, et al. (2011) Prevalence and types of coinfections in sleeping sickness patients in kenya (2000/2009). J Trop Med 2011: 248914.

192. Hira PR, Al-Ali F, Shweiki HM, Abdella NA, Johny M, et al. (2004) Strongyloidiasis: challenges in diagnosis and management in non-endemic Kuwait. Ann Trop Med Parasitol 98: 261-270.

193. Sithithaworn P, Sukavat K, Vannachone B, Sophonphong K, Ben-Embarek P, et al. (2006) Epidemiology of food-borne trematodes and other parasite infections in a fishing community on the Nam Ngum reservoir, Lao PDR. Southeast Asian J Trop Med Public Health 37: 1083-1090.

194. Vannachone B, Kobayashi J, Nambanya S, Manivong K, Inthakone S, et al. (1998) An epidemiological survey on intestinal parasite infection in Khammouane Province, Lao PDR, with special reference to *Strongyloides* infection. Southeast Asian J Trop Med Public Health 29: 717-722.

195. Chai JY, Hongvanthong B (1998) A small-scale survey of intestinal helminthic infections among the residents near Pakse, Laos. Korean J Parasitol 36: 55-58.

196. Sayasone S, Vonghajack Y, Vanmany M, Rasphone O, Tesana S, et al. (2009) Diversity of human intestinal helminthiasis in Lao PDR. Trans R Soc Trop Med Hyg 103: 247-254.

197. Al Kilani MK, Dahesh SM, El Taweel HA (2008) Intestinal parasitosis in Nalout popularity, western Libya. J Egypt Soc Parasitol 38: 255-264.

198. Buchy P (2003) [Intestinal parasitoses in the Mahajanga region, west coast of Madagascar]. Bull Soc Pathol Exot 96: 41-45.

199. Edouard A, Edouard S, Desbois N, Plumelle Y, Rat C, et al. (2004) [Evolution in the prevalence of intestinal parasitosis in the Fort de France University Hospital (Martinique)]. Presse Med 33: 707-709.

200. Gardien E, Schlegel L, Desbois N, Chout R (1997) [Prevalence of intestinal parasitism in the public laboratories of Martinique: development from 1988 to 1995]. Bull Soc Pathol Exot 90: 169-171.

201. Guarner J, Matilde-Nava T, Villasenor-Flores R, Sanchez-Mejorada G (1997) Frequency of intestinal parasites in adult cancer patients in Mexico. Arch Med Res 28: 219-222.

202. Faulkner CT, Garcia BB, Logan MH, New JC, Patton S (2003) Prevalence of endoparasitic infection in children and its relation with cholera prevention efforts in Mexico. Rev Panam Salud Publica 14: 31-41.

203. Mandomando IM, Macete EV, Ruiz J, Sanz S, Abacassamo F, et al. (2007) Etiology of diarrhea in children younger than 5 years of age admitted in a rural hospital of southern Mozambique. Am J Trop Med Hyg 76: 522-527.

204. Evans AC, Joubert JJ (1989) Intestinal helminths of hospital patients in Kavango territory, Namibia. Trans R Soc Trop Med Hyg 83: 681-683.

205. Evans AC, Markus MB, Joubert JJ, Gunders AE (1991) Bushman children infected with the nematode *Strongyloides* *fulleborni*. S Afr Med J 80: 410-411.

206. Evans AC, Markus MB, Steyn E (1990) A survey of the intestinal nematodes of Bushmen in Namibia. Am J Trop Med Hyg 42: 243-247.

207. Hoge CW, Echeverria P, Rajah R, Jacobs J, Malthouse S, et al. (1995) Prevalence of *Cyclospora* species and other enteric pathogens among children less than 5 years of age in Nepal. J Clin Microbiol 33: 3058-3060.

208. Navitsky RC, Dreyfuss ML, Shrestha J, Khatry SK, Stoltzfus RJ, et al. (1998) *Ancylostoma duodenale* is responsible for hookworm infections among pregnant women in the rural plains of Nepal. J Parasitol 84: 647-651.

209. Mukhopadhyay C, Wilson G, Chawla K, Vs B, Shivananda PG (2008) A 6 year Geohelminth infection profile of children at high altitude in Western Nepal. BMC Public Health 8: 98.

210. Tellez A, Morales W, Rivera T, Meyer E, Leiva B, et al. (1997) Prevalence of intestinal parasites in the human population of Leon, Nicaragua. Acta Trop 66: 119-125.

211. Enekwechi LC, Azubike CN (1994) Survey of the prevalence of intestinal parasites in children of primary school age. West Afr J Med 13: 227-230.

212. Agi PI (1995) Pattern of infection of intestinal parasites in Sagbama community of the Niger Delta, Nigeria. West Afr J Med 14: 39-42.

213. Agi PI (1997) Comparative helminth infections of man in two rural communities of the Niger Delta, Nigeria. West Afr J Med 16: 232-236.

214. Dada-Adegbola HO, Bakare RA (2004) Strongyloidiasis in children five years and below. West Afr J Med 23: 194-197.

215. Holland CV, Asaolu SO, Crompton DW, Stoddart RC, Macdonald R, et al. (1989) The epidemiology of *Ascaris lumbricoides* and other soil-transmitted helminths in primary school children from Ile-Ife, Nigeria. Parasitology 99 Pt 2: 275-285.

216. Udonsi JK, Behnke JM, Gilbert FS (1996) Analysis of the prevalence of infection and associations between human gastrointestinal nematodes among different age classes living in the urban and suburban communities of Port Harcourt, Nigeria. J Helminthol 70: 75-84.

217. Akogun OB (1990) Water demand and schistosomiasis among the Gumau people of Bauchi State, Nigeria. Trans R Soc Trop Med Hyg 84: 548-550.

218. Akogun OB (1989) Some social aspects of helminthiasis among the people of Gumau District, Bauchi State, Nigeria. J Trop Med Hyg 92: 193-196.

219. Anosike JC, Nwoke BE, Onwuliri CO, Obiukwu CE, Duru AF, et al. (2004) Prevalence of parasitic diseases among nomadic Fulanis of south-eastern Nigeria. Ann Agric Environ Med 11: 221-225.

220. Anyaeze CM (2003) Reducing burden of hookworm disease in the management of upper abdominal pain in the tropics. Trop Doct 33: 174-175.

221. Onadeko MO, Ladipo OA (1989) Intestinal parasitic infestation in rural communities: a focus for primary health care in Nigeria. Afr J Med Med Sci 18: 289-294.

222. Onwuliri CO, Imandeh NG, Okwuosa VN (1992) Human helminthosis in a rural community of Plateau State, Nigeria. Angew Parasitol 33: 211-216.

223. Sodipo JO, Padgett D, Warrie E, Olopoenia L (1997) Parasitic infections in sickle cell crisis: Nigerian experience. J Natl Med Assoc 89: 285-288.

224. Wagbatsoma VA, Aisien MS (2005) Helminthiasis in selected children seen at the University of Benin Teaching Hospital (UBTH), Benin City, Nigeria. Niger Postgrad Med J 12: 23-27.

225. Wariso BA, Ibe SN (1994) Prevalence of some intestinal helminths in Port Harcourt University of Port Harcourt Teaching Hospital, Nigeria. West Afr J Med 13: 218-222.

226. Ikeh EI, Obadofin MO, Brindeiro B, Baugherb C, Frost F, et al. (2007) Intestinal parasitism in Magama Gumau rural village and Jos township in north central Nigeria. Niger Postgrad Med J 14: 290-295.

227. Agbolade OM, Agu NC, Adesanya OO, Odejayi AO, Adigun AA, et al. (2007) Intestinal helminthiases and schistosomiasis among school children in an urban center and some rural communities in southwest Nigeria. Korean J Parasitol 45: 233-238.

228. Ugbomoiko US, Ofoezie IE (2007) Multiple infection diagnosis of intestinal helminthiasis in the assessment of health and environmental effect of development projects in Nigeria. J Helminthol 81: 227-231.

229. Dada-Adegbola HO, Oluwatoba OA, Bakare RA (2010) Strongyloidiasis: prevalence, risk factors, clinical and laboratory features among diarrhea patients in Ibadan Nigeria. Afr J Med Med Sci 39: 285-292.

230. Abu-Elamreen FH, Abed AA, Sharif FA (2008) Viral, bacterial and parasitic etiology of pediatric diarrhea in Gaza, Palestine. Med Princ Pract 17: 296-301.

231. Patel PK, Khandekar R (2006) Intestinal parasitic infections among school children of the Dhahira Region of Oman. Saudi Med J 27: 627-632.

232. King SE, Mascie-Taylor CG (2004) *Strongyloides* *fuelleborni* *kellyi* and other intestinal helminths in children from Papua New Guinea: associations with nutritional status and socioeconomic factors. P N G Med J 47: 181-191.

233. Yori PP, Kosek M, Gilman RH, Cordova J, Bern C, et al. (2006) Seroepidemiology of strongyloidiasis in the Peruvian Amazon. Am J Trop Med Hyg 74: 97-102.

234. Egido JM, De Diego JA, Penin P (2001) The prevalence of enteropathy due to strongyloidiasis in Puerto Maldonado (Peruvian Amazon). Braz J Infect Dis 5: 119-123.

235. Garcia C, Rodriguez E, Do N, Lopez de Castilla D, Terashima A, et al. (2006) [Intestinal parasitosis in patients with HIV-AIDS]. Rev Gastroenterol Peru 26: 21-24.

236. Rodriguez J, Calderon J (1991) [Intestinal parasitosis in pre-school children from Tarapoto]. Rev Gastroenterol Peru 11: 153-160.

237. Wiedermann U, Stemberger H, Unfried E, Widhalm K, Kundi M, et al. (1991) Intestinal worm burden and serum cholesterol or lipid concentration in a Shipibo population (Peru). Zentralbl Bakteriol 275: 279-286.

238. Roldan WH, Espinoza YA, Huapaya PE, Huiza AF, Sevilla CR, et al. (2009) Frequency of human toxocariasis in a rural population from Cajamarca, Peru determined by DOT-ELISA test. Rev Inst Med Trop Sao Paulo 51: 67-71.

239. Climent C, DeVinatea ML, Lasala G, Ie SO, Velez R, et al. (1994) Geographical pathology profile of AIDS in Puerto Rico: the first decade. Mod Pathol 7: 647-651.

240. Hillyer GV, Soler de Galanes M, Lawrence S (1990) Prevalence of intestinal parasites in a rural community in north-central Puerto Rico. Bol Asoc Med P R 82: 111-114.

241. Lee SK, Shin BM, Chung NS, Chai JY, Lee SH (1994) [Second report on intestinal parasites among the patients of Seoul Paik Hospital (1984-1992)]. Korean J Parasitol 32: 27-33.

242. Youn H (2009) Review of zoonotic parasites in medical and veterinary fields in the Republic of Korea. Korean J Parasitol 47 Suppl: S133-141.

243. Panaitescu D, Capraru T, Bugarin V (1995) Study of the incidence of intestinal and systemic parasitoses in a group of children with handicaps. Roum Arch Microbiol Immunol 54: 65-74.

244. Kurup R, Hunjan GS (2010) Epidemiology and control of Schistosomiasis and other intestinal parasitic infections among school children in three rural villages of south Saint Lucia. J Vector Borne Dis 47: 228-234.

245. al-Madani AA, Mahfouz AA (1995) Prevalence of intestinal parasitic infections among Asian female house keepers in Abha District, Saudi Arabia. Southeast Asian J Trop Med Public Health 26: 135-137.

246. Mohammad KA, Koshak EA (2011) A prospective study on parasites among expatriate workers in Al-Baha from 2009-2011, Saudi Arabia. J Egypt Soc Parasitol 41: 423-432.

247. Al-Megrin WA (2010) Intestinal parasites infection among immunocompromised patients in Riyadh, Saudi Arabia. Pak J Biol Sci 13: 390-394.

248. Bailey MS, Thomas R, Green AD, Bailey JW, Beeching NJ (2006) Helminth infections in British troops following an operation in Sierra Leone. Trans R Soc Trop Med Hyg 100: 842-846.

249. Gbakima AA, Sahr F (1995) Intestinal parasitic infections among rural farming communities in eastern Sierra Leone. Afr J Med Med Sci 24: 195-200.

250. Whitworth JA, Morgan D, Maude GH, McNicholas AM, Taylor DW (1991) A field study of the effect of ivermectin on intestinal helminths in man. Trans R Soc Trop Med Hyg 85: 232-234.

251. Appleton CC, Gouws E (1996) The distribution of common intestinal nematodes along an altitudinal transect in KwaZulu-Natal, South Africa. Ann Trop Med Parasitol 90: 181-188.

252. Appleton CC, Maurihungirire M, Gouws E (1999) The distribution of helminth infections along the coastal plain of Kwazulu-Natal province, South Africa. Ann Trop Med Parasitol 93: 859-868.

253. Diaz J, Igual R, Alonso MC, Moreno MJ (2002) [Intestinal parasitological study in immigrants in the region of Safor (Comunidad Valeciana), Spain]. Med Clin (Barc) 119: 36.

254. Martin Sanchez AM, Hernandez Garcia A, Gonzalez Fernandez M, Afonso Rodriguez O, Hernandez Cabrera M, et al. (2004) [Intestinal parasitosis in the asymptomatic Subsaharian immigrant population. Gran Canaria 2000]. Rev Clin Esp 204: 14-17.

255. Vilalta E, Gascon J, Valls ME, Corachan M (1995) [Ancylostomiasis and strongyloidiasis: clinico-epidemiologic comparative study of travelers coming from endemic areas]. Med Clin (Barc) 105: 292-294.

256. Roman-Sanchez P, Pastor-Guzman A, Moreno-Guillen S, Igual-Adell R, Suner-Generoso S, et al. (2003) High prevalence of *Strongyloides stercoralis* among farm workers on the Mediterranean coast of Spain: analysis of the predictive factors of infection in developed countries. Am J Trop Med Hyg 69: 336-340.

257. Sanchez PR, Guzman AP, Guillen SM, Adell RI, Estruch AM, et al. (2001) Endemic strongyloidiasis on the Spanish Mediterranean coast. QJM 94: 357-363.

258. Marnell F, Guillet A, Holland C (1992) A survey of the intestinal helminths of refugees in Juba, Sudan. Ann Trop Med Parasitol 86: 387-393.

259. Magambo JK, Zeyhle E, Wachira TM (1998) Prevalence of intestinal parasites among children in southern Sudan. East Afr Med J 75: 288-290.

260. Babiker MA, Ali MS, Ahmed ES (2009) Frequency of intestinal parasites among food-handlers in Khartoum, Sudan. East Mediterr Health J 15: 1098-1104.

261. Jozefzoon LM, Oostburg BF (1994) Detection of hookworm and hookworm-like larvae in human fecocultures in Suriname. Am J Trop Med Hyg 51: 501-505.

262. Persson A, Rombo L (1994) Intestinal parasites in refugees and asylum seekers entering the Stockholm area, 1987-88: evaluation of routine stool screening. Scand J Infect Dis 26: 199-207.

263. Tungtrongchitr A, Manatsathit S, Kositchaiwat C, Ongrotchanakun J, Munkong N, et al. (2004) *Blastocystis hominis* infection in irritable bowel syndrome patients. Southeast Asian J Trop Med Public Health 35: 705-710.

264. Anantaphruti MT, Nuamtanong S, Muennoo C, Sanguankiat S, Pubampen S (2000) *Strongyloides stercoralis* infection and chronological changes of other soil-transmitted helminthiases in an endemic area of southern Thailand. Southeast Asian J Trop Med Public Health 31: 378-382.

265. Anantaphruti MT, Waikagul J, Maipanich W, Nuamtanong S, Pubampen S (2004) Soil-transmitted helminthiases and health behaviors among schoolchildren and community members in a west-central border area of Thailand. Southeast Asian J Trop Med Public Health 35: 260-266.

266. Boyajian T (1992) Strongyloidiasis on the Thai-Cambodian border. Trans R Soc Trop Med Hyg 86: 661-662.

267. Jongsuksuntigul P, Intapan PM, Wongsaroj T, Nilpan S, Singthong S, et al. (2003) Prevalence of *Strongyloides stercoralis* infection in northeastern Thailand (agar plate culture detection). J Med Assoc Thai 86: 737-741.

268. Jongwutiwes S, Charoenkorn M, Sitthichareonchai P, Akaraborvorn P, Putaporntip C (1999) Increased sensitivity of routine laboratory detection of *Strongyloides stercoralis* and hookworm by agar-plate culture. Trans R Soc Trop Med Hyg 93: 398-400.

269. Kasuya S, Khamboonruang C, Amano K, Murase T, Araki H, et al. (1989) Intestinal parasitic infections among schoolchildren in Chiang Mai, northern Thailand: an analysis of the present situation. J Trop Med Hyg 92: 360-364.

270. Egger RJ, Hofhuis EH, Bloem MW, Chusilp K, Wedel M, et al. (1990) Association between intestinal parasitoses and nutritional status in 3-8-year-old children in northeast Thailand. Trop Geogr Med 42: 312-323.

271. Khampitak T, Knowles J, Yongvanit P, Sithithaworn P, Tangrassameeprasert R, et al. (2006) Thiamine deficiency and parasitic infection in rural Thai children. Southeast Asian J Trop Med Public Health 37: 441-445.

272. Manatsathit S, Tansupasawasdikul S, Wanachiwanawin D, Setawarin S, Suwanagool P, et al. (1996) Causes of chronic diarrhea in patients with AIDS in Thailand: a prospective clinical and microbiological study. J Gastroenterol 31: 533-537.

273. Nacher M, Singhasivanon P, Treeprasertsuk S, Silamchamroon U, Phumratanaprapin W, et al. (2003) Gender differences in the prevalences of human infection with intestinal helminths on the Thai-Burmese border. Ann Trop Med Parasitol 97: 433-435.

274. Pitisuttithum P, Migasena S, Juntra A, Supeeranond L, Naksrissuk S (1990) Socio-economic status and prevalence of intestinal parasitic infection in Thai adults residing in and around Bangkok metropolis. J Med Assoc Thai 73: 522-525.

275. Punpoowong B, Viriyavejakul P, Riganti M, Pongponaratn E, Chaisri U, et al. (1998) Opportunistic protozoa in stool samples from HIV-infected patients. Southeast Asian J Trop Med Public Health 29: 31-34.

276. Yaicharoen R, Ngrenngarmlert W, Wongjindanon N, Sripochang S, Kiatfuengfoo R (2006) Infection of *Blastocystis hominis* in primary schoolchildren from Nakhon Pathom province, Thailand. Trop Biomed 23: 117-122.

277. Saksirisampant W, Wiwanitkit V, Akrabovorn P, Nuchprayoon S (2002) Parasitic infections in Thai workers that pursue overseas employment: the need for a screening program. Southeast Asian J Trop Med Public Health 33 Suppl 3: 110-112.

278. Supanaranond W, Migasena S, Pitisuttitham P, Suntharasamai P (1990) Health status of Thai volunteers in a cholera vaccine trial. J Med Assoc Thai 73: 548-551.

279. Tungtrongchitr A, Chiworaporn C, Praewanich R, Radomyos P, Boitano JJ (2007) The potential usefulness of the modified Kato thick smear technique in the detection of intestinal sarcocystosis during field surveys. Southeast Asian J Trop Med Public Health 38: 232-238.

280. Waikagul J, Krudsood S, Radomyos P, Radomyos B, Chalemrut K, et al. (2002) A cross-sectional study of intestinal parasitic infections among schoolchildren in Nan Province, Northern Thailand. Southeast Asian J Trop Med Public Health 33: 218-223.

281. Waree P, Polseela P, Pannarunothai S, Pipitgool V (2001) The present situation of paragonimiasis in endemic area in Phitsanulok Province. Southeast Asian J Trop Med Public Health 32 Suppl 2: 51-54.

282. Waywa D, Kongkriengdaj S, Chaidatch S, Tiengrim S, Kowadisaiburana B, et al. (2001) Protozoan enteric infection in AIDS related diarrhea in Thailand. Southeast Asian J Trop Med Public Health 32 Suppl 2: 151-155.

283. Wilairatana P, Radomyos P, Radomyos B, Phraevanich R, Plooksawasdi W, et al. (1996) Intestinal sarcocystosis in Thai laborers. Southeast Asian J Trop Med Public Health 27: 43-46.

284. Wiwanitkit V (2001) Intestinal parasitic infections in Thai HIV-infected patients with different immunity status. BMC Gastroenterol 1: 3.

285. Wongjindanon N, Suksrichavalit T, Subsutti W, Sarachart T, Worapisuttiwong U, et al. (2005) Current infection rate of *Giardia* *lamblia* in two provinces of Thailand. Southeast Asian J Trop Med Public Health 36 Suppl 4: 21-25.

286. Yaicharoen R, Sripochang S, Sermsart B, Pidetcha P (2005) Prevalence of *Blastocystis hominis* infection in asymptomatic individuals from Bangkok, Thailand. Southeast Asian J Trop Med Public Health 36 Suppl 4: 17-20.

287. Nuchprayoon S, Siriyasatien P, Kraivichian K, Porksakorn C, Nuchprayoon I (2002) Prevalence of parasitic infections among Thai patients at the King Chulalongkorn Memorial Hospital, Bangkok, Thailand. J Med Assoc Thai 85 Suppl 1: S415-423.

288. Saksirisampant W, Nuchprayoon S, Wiwanitkit V, Yenthakam S, Ampavasiri A (2003) Intestinal parasitic infestations among children in an orphanage in Pathum Thani province. J Med Assoc Thai 86 Suppl 2: S263-270.

289. Koga K, Kasuya S, Khamboonruang C, Sukavat K, Nakamura Y, et al. (1990) An evaluation of the agar plate method for the detection of *Strongyloides stercoralis* in northern Thailand. J Trop Med Hyg 93: 183-188.

290. Koga K, Kasuya S, Khamboonruang C, Sukhavat K, Ieda M, et al. (1991) A modified agar plate method for detection of *Strongyloides stercoralis*. Am J Trop Med Hyg 45: 518-521.

291. Nacher M, Singhasivanon P, Yimsamran S, Manibunyong W, Thanyavanich N, et al. (2002) Intestinal helminth infections are associated with increased incidence of *Plasmodium falciparum* malaria in Thailand. J Parasitol 88: 55-58.

292. Nontasut P, Muennoo C, Sa-nguankiat S, Fongsri S, Vichit A (2005) Prevalence of *strongyloides* in Northern Thailand and treatment with ivermectin vs albendazole. Southeast Asian J Trop Med Public Health 36: 442-444.

293. Sithithaworn P, Srisawangwong T, Tesana S, Daenseekaew W, Sithithaworn J, et al. (2003) Epidemiology of *Strongyloides stercoralis* in north-east Thailand: application of the agar plate culture technique compared with the enzyme-linked immunosorbent assay. Trans R Soc Trop Med Hyg 97: 398-402.

294. Sithithaworn J, Sithithaworn P, Janrungsopa T, Suvatanadecha K, Ando K, et al. (2005) Comparative assessment of the gelatin particle agglutination test and an enzyme-linked immunosorbent assay for diagnosis of strongyloidiasis. J Clin Microbiol 43: 3278-3282.

295. Sukhavat K, Morakote N, Chaiwong P, Piangjai S (1994) Comparative efficacy of four methods for the detection of *Strongyloides stercoralis* in human stool specimens. Ann Trop Med Parasitol 88: 95-96.

296. Uparanukraw P, Phongsri S, Morakote N (1999) Fluctuations of larval excretion in *Strongyloides stercoralis* infection. Am J Trop Med Hyg 60: 967-973.

297. Pinlaor S, Mootsikapun P, Pinlaor P, Pipitgool V, Tuangnadee R (2005) Detection of opportunistic and non-opportunistic intestinal parasites and liver flukes in HIV-positive and HIV-negative subjects. Southeast Asian J Trop Med Public Health 36: 841-845.

298. Viriyavejakul P, Nintasen R, Punsawad C, Chaisri U, Punpoowong B, et al. (2009) High prevalence of *Microsporidium* infection in HIV-infected patients. Southeast Asian J Trop Med Public Health 40: 223-228.

299. Warunee N, Choomanee L, Sataporn P, Rapeeporn Y, Nuttapong W, et al. (2007) Intestinal parasitic infections among school children in Thailand. Trop Biomed 24: 83-88.

300. Kitvatanachai S, Boonslip S, Watanasatitarpa S (2008) Intestinal parasitic infections in Srimum suburban area of Nakhon Ratchasima Province, Thailand. Trop Biomed 25: 237-242.

301. Ayadi A, Mahjoubi F, Makni F (1992) [Intestinal parasitism in the adult. Evaluation of 2 years in the University Hospital Center of Sfax]. Bull Soc Pathol Exot 85: 44-46.

302. Culha G (2006) [The distribution of patients with intestinal parasites presenting at the parasitology laboratory of the Mustafa Kemal University medical faculty]. Turkiye Parazitol Derg 30: 302-304.

303. Alver O, Ozakin C, Yilmaz E, Akcaglar S, Tore O (2005) [Evaluation of the distribution of intestinal parasites in the Uludag University Medical Faculty during a period of eight years.]. Turkiye Parazitol Derg 29: 193-199.

304. Buyukbaba Boral O, Uysal H, Alan S, Nazlican O (2004) [Investigation of intestinal parasites in AIDS patients]. Mikrobiyol Bul 38: 121-128.

305. Brown M, Bukusuba J, Hughes P, Nakiyingi J, Watera C, et al. (2003) Screening for intestinal helminth infestation in a semi-urban cohort of HIV-infected people in Uganda: a combination of techniques may enhance diagnostic yield in the absence of multiple stool samples. Trop Doct 33: 72-76.

306. Kabatereine NB, Kemijumbi J, Kazibwe F, Onapa AW (1997) Human intestinal parasites in primary school children in Kampala, Uganda. East Afr Med J 74: 311-314.

307. Lin CJ, Katongole-Mbidde E, Byekwaso T, Orem J, Rabkin CS, et al. (2008) Intestinal parasites in Kaposi sarcoma patients in Uganda: indication of shared risk factors or etiologic association. Am J Trop Med Hyg 78: 409-412.

308. Muhangi L, Woodburn P, Omara M, Omoding N, Kizito D, et al. (2007) Associations between mild-to-moderate anaemia in pregnancy and helminth, malaria and HIV infection in Entebbe, Uganda. Trans R Soc Trop Med Hyg 101: 899-907.

309. Stothard JR, Pleasant J, Oguttu D, Adriko M, Galimaka R, et al. (2008) *Strongyloides stercoralis*: a field-based survey of mothers and their preschool children using ELISA, Baermann and Koga plate methods reveals low endemicity in western Uganda. J Helminthol 82: 263-269.

310. Sousa-Figueiredo JC, Day M, Betson M, Rowell C, Wamboko A, et al. (2011) Field survey for strongyloidiasis in eastern Uganda with observations on efficacy of preventive chemotherapy and co-occurrence of soil-transmitted helminthiasis/intestinal schistosomiasis. J Helminthol 85: 325-333.

311. Gill GV, Welch E, Bailey JW, Bell DR, Beeching NJ (2004) Chronic *Strongyloides stercoralis* infection in former British Far East prisoners of war. QJM 97: 789-795.

312. Tarimo DS, Killewo JZ, Minjas JN, Msamanga GI (1996) Prevalence of intestinal parasites in adult patients with enteropathic AIDS in north-eastern Tanzania. East Afr Med J 73: 397-399.

313. Gomez Morales MA, Atzori C, Ludovisi A, Rossi P, Scaglia M, et al. (1995) Opportunistic and non-opportunistic parasites in HIV-positive and negative patients with diarrhoea in Tanzania. Trop Med Parasitol 46: 109-114.

314. Range N, Magnussen P, Mugomela A, Malenganisho W, Changalucha J, et al. (2007) HIV and parasitic co-infections in tuberculosis patients: a cross-sectional study in Mwanza, Tanzania. Ann Trop Med Parasitol 101: 343-351.

315. Dreyfuss ML, Msamanga GI, Spiegelman D, Hunter DJ, Urassa EJ, et al. (2001) Determinants of low birth weight among HIV-infected pregnant women in Tanzania. Am J Clin Nutr 74: 814-826.

316. Knopp S, Mohammed KA, Simba Khamis I, Mgeni AF, Stothard JR, et al. (2008) Spatial distribution of soil-transmitted helminths, including *Strongyloides stercoralis*, among children in Zanzibar. Geospat Health 3: 47-56.

317. Knopp S, Mgeni AF, Khamis IS, Steinmann P, Stothard JR, et al. (2008) Diagnosis of soil-transmitted helminths in the era of preventive chemotherapy: effect of multiple stool sampling and use of different diagnostic techniques. PLoS Negl Trop Dis 2: e331.

318. Salazar SA, Gutierrez C, Berk SL (1995) Value of the agar plate method for the diagnosis of intestinal strongyloidiasis. Diagn Microbiol Infect Dis 23: 141-145.

319. Schupf N, Ortiz M, Kapell D, Kiely M, Rudelli RD (1995) Prevalence of intestinal parasite infections among individuals with mental retardation in New York State. Ment Retard 33: 84-89.

320. Safdar A, Malathum K, Rodriguez SJ, Husni R, Rolston KV (2004) Strongyloidiasis in patients at a comprehensive cancer center in the United States. Cancer 100: 1531-1536.

321. Lurio J, Verson H, Karp S (1991) Intestinal parasites in Cambodians: comparison of diagnostic methods used in screening refugees with implications for treatment of populations with high rates of infestation. J Am Board Fam Pract 4: 71-78.

322. Buchwald D, Lam M, Hooton TM (1995) Prevalence of intestinal parasites and association with symptoms in Southeast Asian refugees. J Clin Pharm Ther 20: 271-275.

323. Cartwright CP (1999) Utility of multiple-stool-specimen ova and parasite examinations in a high-prevalence setting. J Clin Microbiol 37: 2408-2411.

324. Ciesielski SD, Seed JR, Ortiz JC, Metts J (1992) Intestinal parasites among North Carolina migrant farmworkers. Am J Public Health 82: 1258-1262.

325. Kitchen LW, Tu KK, Kerns FT (2000) *Strongyloides*-infected patients at Charleston area medical center, West Virginia, 1997-1998. Clin Infect Dis 31: E5-6.

326. Wehner JH, Kirsch CM, Kagawa FT, Jensen WA, Campagna AC, et al. (1994) The prevalence and response to therapy of *Strongyloides stercoralis* in patients with asthma from endemic areas. Chest 106: 762-766.

327. Garg PK, Perry S, Dorn M, Hardcastle L, Parsonnet J (2005) Risk of intestinal helminth and protozoan infection in a refugee population. Am J Trop Med Hyg 73: 386-391.

328. Geltman PL, Cochran J, Hedgecock C (2003) Intestinal parasites among African refugees resettled in Massachusetts and the impact of an overseas pre-departure treatment program. Am J Trop Med Hyg 69: 657-662.

329. Kappus KK, Juranek DD, Roberts JM (1991) Results of testing for intestinal parasites by state diagnostic laboratories, United States, 1987. MMWR CDC Surveill Summ 40: 25-45.

330. Lifson AR, Thai D, O'Fallon A, Mills WA, Hang K (2002) Prevalence of tuberculosis, hepatitis B virus, and intestinal parasitic infections among refugees to Minnesota. Public Health Rep 117: 69-77.

331. Miller JM, Boyd HA, Ostrowski SR, Cookson ST, Parise ME, et al. (2000) Malaria, intestinal parasites, and schistosomiasis among Barawan Somali refugees resettling to the United States: a strategy to reduce morbidity and decrease the risk of imported infections. Am J Trop Med Hyg 62: 115-121.

332. Seybolt LM, Christiansen D, Barnett ED (2006) Diagnostic evaluation of newly arrived asymptomatic refugees with eosinophilia. Clin Infect Dis 42: 363-367.

333. Ribes JA, Seabolt JP, Overman SB (2004) Point prevalence of *Cryptosporidium*, *Cyclospora*, and *Isospora* infections in patients being evaluated for diarrhea. Am J Clin Pathol 122: 28-32.

334. Lillie PJ, Bazaz R, Greig JM (2008) Screening African HIV positive patients for imported parasitic infections. J Infect 57: 481-484.

335. Posey DL, Blackburn BG, Weinberg M, Flagg EW, Ortega L, et al. (2007) High prevalence and presumptive treatment of schistosomiasis and strongyloidiasis among African refugees. Clin Infect Dis 45: 1310-1315.

336. Brodine SK, Thomas A, Huang R, Harbertson J, Mehta S, et al. (2009) Community based parasitic screening and treatment of Sudanese refugees: application and assessment of Centers for Disease Control guidelines. Am J Trop Med Hyg 80: 425-430.

337. Hochberg NS, Moro RN, Sheth AN, Montgomery SP, Steurer F, et al. (2011) High prevalence of persistent parasitic infections in foreign-born, HIV-infected persons in the United States. PLoS Negl Trop Dis 5: e1034.

338. Arenas-Pinto A, Certad G, Ferrara G, Castro J, Bello MA, et al. (2003) Association between parasitic intestinal infections and acute or chronic diarrhoea in HIV-infected patients in Caracas, Venezuela. Int J STD AIDS 14: 487-492.

339. Chacin-Bonilla L, Guanipa N, Cano G, Raleigh X, Quijada L (1992) Cryptosporidiosis among patients with acquired immunodeficiency syndrome in Zulia State, Venezuela. Am J Trop Med Hyg 47: 582-586.

340. Miller SA, Rosario CL, Rojas E, Scorza JV (2003) Intestinal parasitic infection and associated symptoms in children attending day care centres in Trujillo, Venezuela. Trop Med Int Health 8: 342-347.

341. Le Hung Q, de Vries PJ, Giao PT, Binh TQ, Nam NV, et al. (2005) Intestinal helminth infection in an ethnic minority commune in southern Vietnam. Southeast Asian J Trop Med Public Health 36: 623-628.

342. Graczyk TK, Shiff CK, Tamang L, Munsaka F, Beitin AM, et al. (2005) The association of *Blastocystis hominis* and *Endolimax nana* with diarrheal stools in Zambian school-age children. Parasitol Res 98: 38-43.

343. Conlon CP, Pinching AJ, Perera CU, Moody A, Luo NP, et al. (1990) HIV-related enteropathy in Zambia: a clinical, microbiological, and histological study. Am J Trop Med Hyg 42: 83-88.

344. Hunter G, Bagshawe AF, Baboo KS, Luke R, Prociv P (1992) Intestinal parasites in Zambian patients with AIDS. Trans R Soc Trop Med Hyg 86: 543-545.
