# Supplementary material for: Strongyloides stercoralis: Global Distribution and Risk Factors
Source: PLoS Negl Trop Dis. 2013 Jul 11;7(7):e2288. doi: 10.1371/journal.pntd.0002288 (PMC3708837; doi:10.1371/journal.pntd.0002288)
Supplement: Text S1 — Appendix: Estimation of country-specific prevalence and estimation of prevalence in specific risk groups. (DOC) [file pntd.0002288.s004.doc]

**Appendix**

**Estimation of country-specific prevalence**

A binomial model was used to estimate prevalence rates taking into account the number of individuals screened, the number of positive individuals and the sensitivity coefficient of the diagnostic test used. The model was formulated and implemented under a Bayesian framework.

Let $Y_{ijk}$ denote the number of positive samples out of $N_{ijk}$ individuals screened using a diagnostic tool of sensitivity category $k$ ($k=1,2,3$) in country $i,\text{ }i=1,...,C$ and study $j=1,...,n_{i}$, where $n_{i}$ is the total number of surveys in country $i$ and $C=236$ is the total number of countries analysed. The model assumes that $Y_{ijk}$ is distributed binomially, that is:, where $p_{ijk}$ is the observed prevalence of *S. stercoralis*. If $D_{i}+$ denotes an individual who is truly infected by *S. stercoralis* in country $i$, the observed prevalence$p_{ijk}$ is related to the true prevalence through the equation $(1)$

where $T_{ijk}+$ denotes that an individual in country $i$, survey $j$ has tested positive using a diagnostic tool with sensitivity category $k$. Let $\pi_{i}$ indicate the true prevalence, i.e. $\pi_{i}=P(D_{i}+)$, equation (1) can be written as $p_{ijk}=S_{ijk}\pi_{i}$ $(2)$

where $S_{ijk}=P(T_{ijk}+|D_{i}+)$ is the sensitivity value of the diagnostic tool used in study $j$. Assuming that only one diagnostic tool was used in each study $j$ and that its sensitivity depends only on the category (low, moderate or high), equation (2) becomes $p_{ij}=S_{k}\pi_{i}$.

Since actual prior data for prevalence were unavailable, an uninformative prior was elicited on $\pi_{i}$,i.e. $\pi_{i}\sim Beta(a,b)$.Several choices of the hyperparameters $a$ and $b$ were implemented, with the aim of conducting a probabilistic sensitivity analysis, leading to very similar estimates. The hyperparameters $a$ and $b$ where chosen in order to define a unimodal distribution with large variance.

Based on information found in the literature, the prior distributions on the sensitivity of the diagnostic tools of category low, moderate and high were specified as follows: $S_{1}\sim Beta(1,1)I(\text{0.129,0.689})$,$S_{2}\sim Beta(1,1)I(\text{0.471,0.968})$,$S_{3}\sim Beta(1,1)I(\text{0.68,0.982})$.

**Estimation of prevalence in specific risk groups**

For the second analysis, the same prior information on the sensitivity of diagnostic tools was used. The model was formulated in the odds ratio scale, considering each risk group separately. Let model the distribution of infected individuals in risk group *r* and $Y_{jk}^{c}$ denote the one in the control group, i.e. ,

a logistic regression equation can be written on the risks $\text{logit}(p_{jk}^{c})=\mu_{jk}^{c}$, $\text{logit}(p_{jk}^{r})=\mu_{jk}^{c}+\delta_{jk}^{r}$ where a prior distribution is given on the $\delta_{jk}^{r}\sim N(c,d)$, where the latter represents the risk effect in the scale relative to the control group. For each risk group a separate regression model was fitted.

The results of the models were expressed in terms of posterior medians and Bayesian Credible Intervals.

**Table A1: Posterior diagnostic test sensitivity estimates: community-based studies**

| **Sensitivity** | **Prior** | **Posterior**  **(median, 95% BCI)** | **Prior** | **Posterior (median, 95% BCI)** |
| --- | --- | --- | --- | --- |
| 1. Low | U(0.13-0.69) | 0.17, (0.15-0.18) | Beta(3.7,5.69) | 0.17, (0.15-0.18) |
| 2. Moderate | U(0.47-0.97) | 0.84, (0.77-0.90) | Beta(7.13,2.54) | 0.84, (0.78-0.90) |
| 3. High | U(0.68-0.98) | 0.95, (0.88-0.98) | Beta(10.76,2.09) | 0.95, (0.90-0.98) |

**Table A2: Posterior diagnostic test sensitivity estimates: studies among immigrants.**

| **Sensitivity** | **Prior** | **Posterior**  **(median, 95% BCI)** | **Prior** | **Posterior (median, 95% BCI)** |
| --- | --- | --- | --- | --- |
| 1. Low | U(0.13-0.69) | 0.13, (0.13-0.14) | Beta(3.7,5.69) | 0.15, (0.15-0.16) |
| 2. Moderate | U(0.47-0.97) | 0.70, (0.49-0.95) | Beta(7.13,2.54) | 0.73, (0.52-0.95) |
| 3. High | U(0.68-0.98) | 0.97, (0.94-0.98) | Beta(10.76,2.09) | 0.97, (0.94-0.98) |

**Table A3: Posterior diagnostic test sensitivity estimates: hospital-based studies.**

| **Sensitivity** | **Prior** | **Posterior (median, 95% BCI)** | **Prior** | **Posterior (median, 95% BCI)** |
| --- | --- | --- | --- | --- |
| 1. Low | U(0.13-0.69) | 0.19, (0.17-0.21) | Beta(3.7,5.69) | 0.20, (0.20-0.22) |
| 2. Moderate | U(0.47-0.97) | 0.47, (0.47-0.48) | Beta(7.13,2.54) | 0.50, (0.48-0.51) |
| 3. High | U(0.68-0.98) | 0.98, (0.97-0.98) | Beta(10.76,2.09) | 0.98, (0.97-0.98) |

**Figure Captures**

Figure A1a: Risk of *S. stercoralis* infection in HIV/AIDS patients (meta-analysis of case-control studies, excluding sensitivity of diagnostic test)

Figure A1b: Risk of *S. stercoralis* infection in patients with HTLV-1 infection (meta-analysis of case-control studies, excluding sensitivity of diagnostic test)

Figure A1c: Risk of *S. stercoralis* infection in alcoholics (meta-analysis of case-control studies, excluding sensitivity of diagnostic test)

Figure A1d: Risk of *S. stercoralis* infection in patients with diarrhoea (meta-analysis of case-control studies, excluding sensitivity of diagnostic test)

**Figure A1a**

**Figure A1b**

**Figure A1c**

**Figure A1d**
